# Effect of OASL on oxaliplatin-induced immunogenic cell death in gastric cancer via the cGAS-STING signaling pathway

**DOI:** 10.1038/s41420-025-02850-w

**Published:** 2025-11-21

**Authors:** Lingling Zhang, Yi Liu, Haiying Yang, Luguang Liu, Longgang Wang, Jie Chai, Weizhu Zhao, Dong Sun

**Affiliations:** 1 Department of Oncology, Binzhou People’s Hospital Affiliated to Shandong First Medical University, Binzhou, Shandong China; 2 Department of Pain, Binzhou People’s Hospital Affiliated to Shandong First Medical University, Binzhou, Shandong China; 3 Department of Gastroenterological surgery, Shandong Cancer Hospital and Institute, Shandong First Medical University and Shandong Academy of Medical Sciences, Shandong Cancer Hospital Affiliated to Shandong First Medical University, Jinan, Shandong China; 4 Key Discipline of Medicine and Health in Shandong Province (Department of General Surgery, Binzhou People’s Hospital), Binzhou, Shandong China

**Keywords:** Cancer microenvironment, Gastrointestinal diseases

## Abstract

This study investigates the role of 2’-5’ oligoadenylate synthetase-like (OASL) in Oxaliplatin (OXA)-induced immunogenic cell death (ICD) in Gastric cancer (GC) cells through the cGAS-STING signaling pathway. Knockdown of OASL enhanced ICD expression, while overexpression had the opposite effect. mRNA sequencing of OASL-knockdown and control GC cells treated with OXA revealed significant enrichment of the cGAMP-mediated second messenger signaling pathway. cGAS synthesizes the second messenger molecule cGAMP, which directly activated STING. To clarify the mechanism, the role of OASL in OXA-induced ICD in GC cells was validated as mediated by the cGAS-STING signaling pathway. The Co-IP and immunofluorescence results confirmed that the OASL and cGAS proteins can bind directly. Further research using an in vivo mouse model validated these findings. Results show that OASL regulates OXA-induced ICD in GC cells via the cGAS-STING pathway, impacting chemosensitivity. The findings suggest new targets and strategies for improving GC therapy by modulating OASL expression to enhance OXA sensitivity through ICD mechanisms.

## Introduction

Gastric cancer (GC) is the fifth most common malignancy worldwide, ranking as the fourth leading cause of cancer-related deaths, and is one of the most common malignant tumors in China [1, 2]. Despite recent progress in basic and clinical research on GC, with novel chemotherapeutic agents continually introduced to clinical practice and therapeutic approaches such as cancer immunotherapy and targeted therapy constantly evolving, the prognosis for GC patients remains poor [3–6]. Chemotherapy plays an indispensable role in advanced GC [7]. Although oxaliplatin(OXA)-based chemotherapy regimens can significantly improve the prognosis of patients with GC, the clinical response rate is only approximately 40-67% [8, 9]. Furthermore, it has been found that OXA is often prone to severe drug resistance during clinical treatment. Previous studies have shown that altering the expression of certain molecules in GC cells can improve their sensitivity to OXA chemotherapy. Mechanisms of resistance to OXA in GC involve the coordinated regulation of multiple pathways. Studies have shown that JUNB enhances resistance by activating the MAPK signaling pathway, and combining it with ERK/MEK inhibitors can reverse resistance [10]. EphA2 reduces drug sensitivity by inducing EMT, and its silencing can restore cell response to OXA [11]. At the epigenetic level, METTL3 maintains the resistant characteristics of CD133+ stem cells through dual mechanisms: promoting DNA repair (base excision repair) and maintaining PARP1 mRNA stability [12, 13]. Research on autophagy indicates that the LINC00641/miR-582-5p axis promotes resistance by activating autophagy, and inhibiting this axis can enhance the toxicity of OXA [14].However, these indicators have not yet been widely used in clinical practice [15, 16]. Therefore, it is important to conduct basic GC research at multiple levels to further clarify the molecular mechanisms involved in the development of GC.

The 2′-5′ oligoadenylate synthetase (OAS) gene is one of the interferon-stimulated genes. The OAS family consists of four members: OAS1, OAS2, OAS3, and ubiquitin-like 2′-5′ oligoadenylate synthetase (OASL). Unlike the others, OASL contains an oligoadenylate-like domain at its N terminus but lacks oligoadenylate synthetase activity. It also has two ubiquitin-like domains at its C terminus, which are critical for OASL to exert its antiviral activity [17–20]. Current studies have found that OASL plays an important role in immune regulation and maintenance of drug metabolism [21–23]. A recent study found that the correlation between OASL and cancer has been gradually confirmed, which may participate in the development of breast cancer [24], pancreatic cancer [25], and cervical cancer [26], and is closely related to prognosis. Our previous study also found that OASL promotes the proliferation, invasion, and migration of GC cells and inhibits their apoptosis, and that OASL functional as an oncogene in the development of GC [27]. OASL is a decisive regulator in maintaining the sensitivity of lung cancer cells to acRoots, which may be related to the development of drug resistance. Modulation of OASL could be an alternative strategy for improving drug efficacy during cancer treatment [28]. OAS family members have all been identified as central genes in trastuzumab-resistant GC and controls [29], suggesting that OASL also plays an important role in maintaining sensitivity to drug therapy in GC. Recent studies have shown that OXA plays an important role in addition to cytotoxicity and that its anti-tumor activity is closely related to the regulation and improvement of immune function [30]. The specific immunological mechanisms of anti-tumor activity are: the induction of immunogenic cell death (ICD), the effect on the STAT protein signaling pathway, and regulation of the tumor microenvironment [31–35]. Accordingly, we speculated that OASL may play an important role in maintaining OXA chemotherapy sensitivity in GC.

Therefore, this study began with the anti-tumor immunological mechanism of OXA. GC cells were treated with OXA after OASL knockdown or overexpression. ICD expression was enhanced after OASL knockdown, whereas OASL overexpression had the opposite effect. OASL knockdown of GC cells and common GC cells were administered OXA for 24 h for mRNA sequencing, which significantly enriched the second messenger signaling pathway (cGAMP). This also indicates that OASL can regulate OXA-induced ICD in GC cells through the cGAS-STING signaling pathway, thus affecting chemosensitivity. The results of this study provide new targets and strategies for GC therapy.

## Results

### Oxaliplatin is able to induce immunogenic cell death in gastric cancer cells

#### Effect of oxaliplatin on the proliferation and apoptosis of gastric cancer cells

**Fig. 1:**
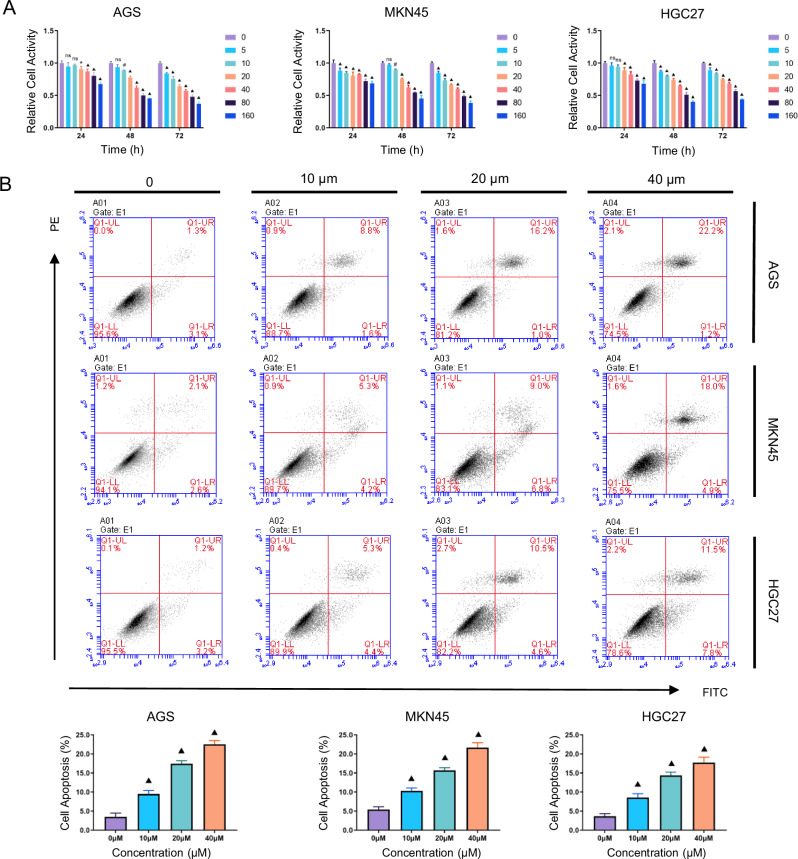
Effect of OXA on the proliferation and apoptosis of GC cells. **A** AGS, MKN45, and HGC27 cells were treated with OXA at various concentrations (0, 5, 10, 20, 40, 80, and 160 μM) for 24, 48, and 72 h. The proliferation of GC cells was assessed using the CCK-8 assay. **B** AGS, MKN45, and HGC27 cells were treated with OXA at various concentrations (0, 10, 20, and 40 μM) for 48 h. The apoptosis rates of GC cells were measured using flow cytometry with the Annexin-FITC/PI double staining method. The values indicate the mean ± standard deviation (SD) of three independent experiments. Statistical significance is indicated as follows: “ns” indicates no statistically significant difference, “*” indicates *P* < 0.05, “#“ indicates *P* < 0.01, and “▲” indicates *P* < 0.001.

To examine the effects of OXA on GC cells, AGS, MKN45, and HGC27 cells were treated with increasing OXA concentrations (0, 5, 10, 20, 40, 80, and 160 μM) for 24, 48, and 72 h. CCK-8 proliferation assays demonstrated significant time- and dose-dependent growth inhibition (Fig. 1A). Parallel apoptosis analysis revealed that 48-hour exposure to OXA (0, 10, 20, and 40 μM) dose-dependently increased apoptotic rates as quantified by Annexin V-FITC/PI dual staining flow cytometry (*P* < 0.001; Fig. 1B).

#### Oxaliplatin induces immunogenic cell death in gastric cancer cells

**Fig. 2:**
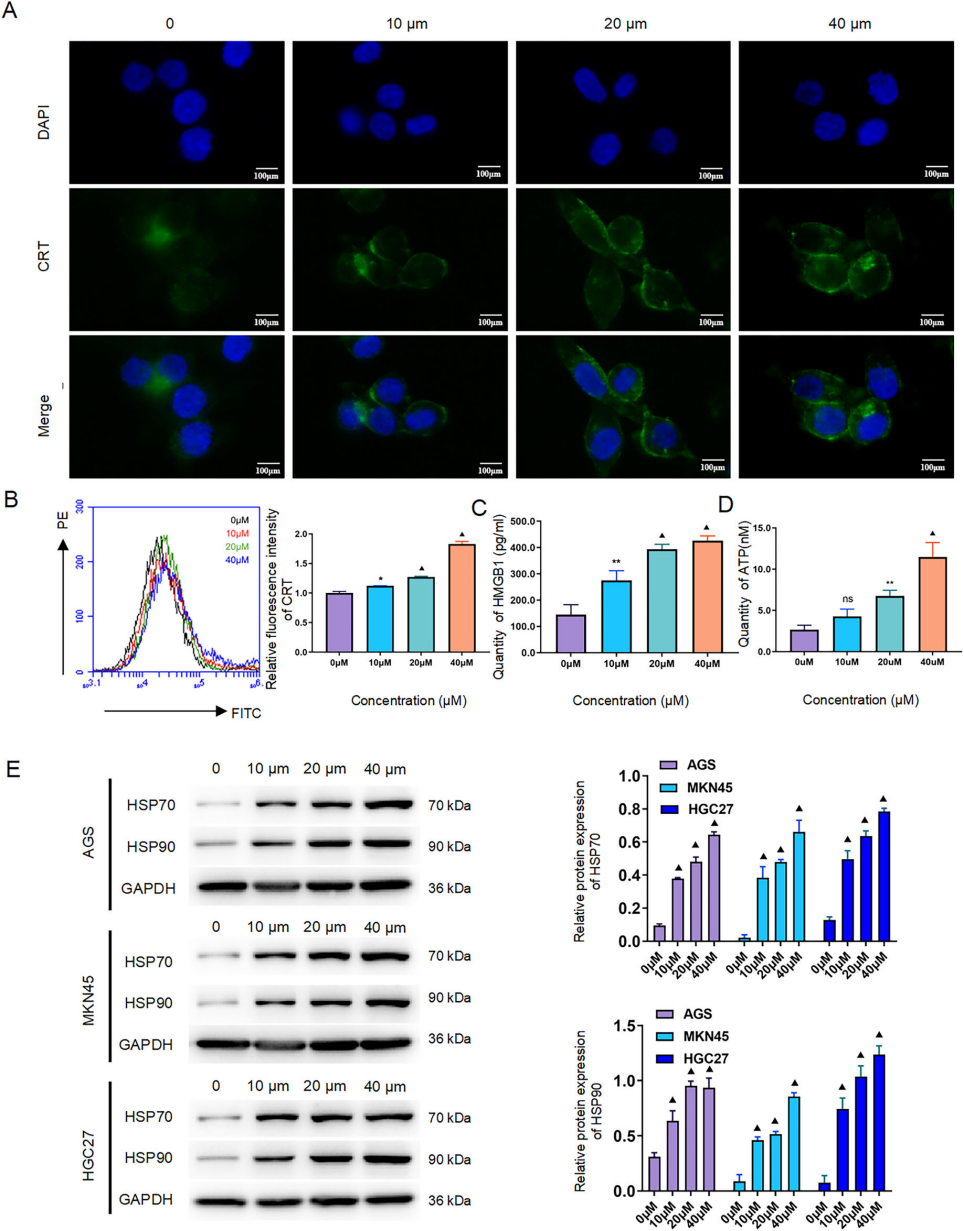
OXA induces immunogenic cell death in GC cells. **A** AGS cells were treated with OXA at various concentrations (0, 10, 20, and 40 μM) for 48 hours. The changes in CRT were observed via immunofluorescence. **B** AGS cells were treated with OXA at various concentrations (0, 10, 20, and 40 μM) for 48 h. The expression level of CRT on the cell membrane was measured using flow cytometry with dead cells, and a statistical chart was generated. **C** AGS cells were treated with OXA at various concentrations (0, 10, 20, and 40 μM) for 48 hours. The content of HMGB1 in the cell supernatant was measured using ELISA, and a statistical chart was generated. **D** AGS cells were treated with OXA at concentrations (0, 10, 20, and 40 μM) for 48 h. The ATP content in the supernatant was quantified using an ATP assay kit, and a statistical chart was generated. **E** AGS, MKN45, and HGC27 cells were treated with OXA at various concentrations (0, 10, 20, and 40 μM) for 48 hours. Western blot analysis was performed to assess the expression levels of HSP70 and HSP90 proteins, and a statistical chart was generated. The values indicate the mean ± standard deviation (SD) of three independent experiments. Scale bar: 100 μm. Statistical significance is indicated as follows: “ns” indicates no statistically significant difference, “*” indicates *P* < 0.05,”**“ indicates *P* < 0.01, and “▲” indicates *P* < 0.001.

Immunogenic cell death (ICD) is characterized by damage-associated molecular pattern (DAMP) release, including calreticulin (CRT) surface exposure, HMGB1 translocation, and ATP secretion. To evaluate OXA-induced ICD in GC cells, AGS, MKN45, and HGC27 cells were treated with increasing OXA concentrations (0, 10, 20, and 40 μM) for 48 hours. Immunofluorescence analysis demonstrated dose-dependent CRT translocation from the cytoplasm to the plasma membrane (Fig. 2A, B; Supplementary Fig. S1 and S2).

Concomitantly, ELISA quantification of cell supernatants revealed significantly elevated extracellular HMGB1 (*P* < 0.01) and ATP (*P* < 0.001) levels in OXA-treated groups versus controls (Fig. 2C, D). Western blot analysis further confirmed dose-responsive upregulation of HSP70 and HSP90 proteins across all treatment concentrations (*P* < 0.001; Fig. 2E).

### OASL is able to inhibit oxaliplatin-induced immunogenic cell death in gastric cancer cells

#### Effect of OASL on oxaliplatin-induced apoptosis in gastric cancer cells

**Fig. 3:**
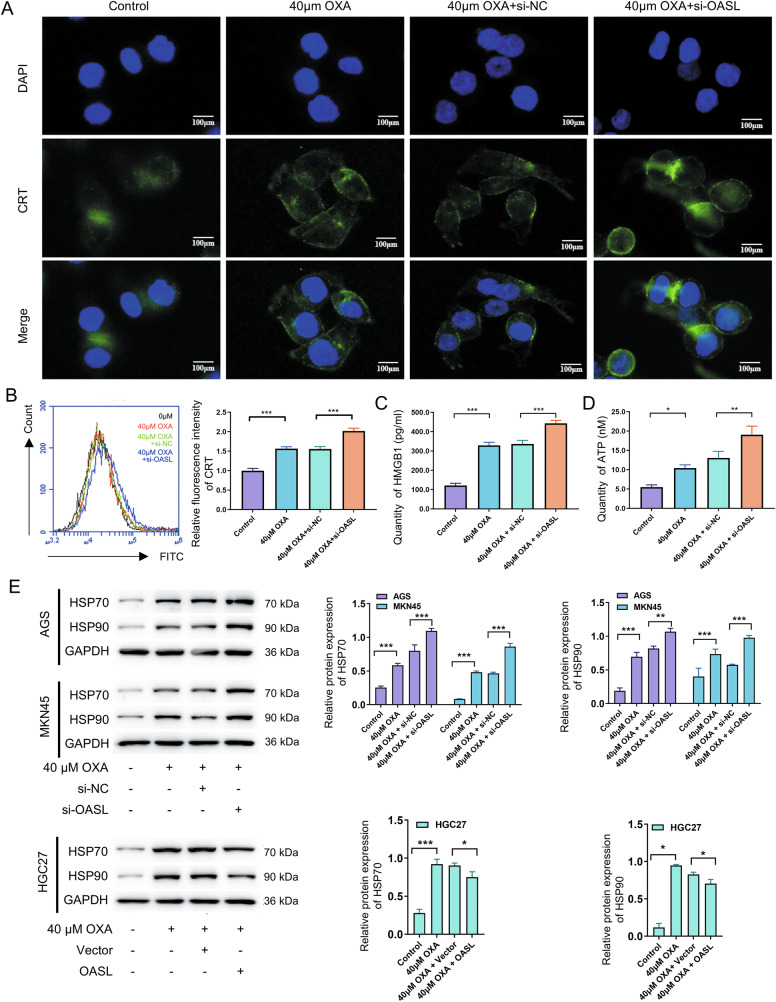
The Effect of OASL on OXA-Induced apoptosis in GC cells. **A** AGS, MKN45, and HGC27 cells were treated with OXA at various concentrations (0, 10, 20, and 40 μM) for 48 h. The Western blot analysis was performed to assess the protein expression levels of OASL in GC cells, followed by statistical analysis. **B** AGS, MKN45, and HGC27 cells were treated with OXA at various concentrations (0, 10, 20, and 40 μM) for 48 h. RT-PCR was then utilized to measure OASL mRNA expression levels in the GC cells, followed by statistical analysis. **C** AGS cells underwent OASL knockdown and were subsequently treated with OXA at different concentrations (0, 10, 20, and 40 μM) for 48 h. The apoptosis rate was measured by flow cytometry using the Annexin FITC/PI double staining method, and a statistical analysis of the apoptosis rate was conducted. **D** HGC27 cells were overexpressed with OASL and then treated with 40 μM OXA for 48 h. Flow cytometry (using the Annexin FITC/PI double staining method) was employed to measure the apoptosis rate of AGS cells, along with the generation of a statistical chart reflecting the apoptosis rate. **E** Statistical graphs showed LDH content in AGS and MKN45 cells after OASL knockdown, followed by treatment with OXA at various concentrations (0, 10, 20, and 40 μM) for 48 h, as well as in HGC27 cells after OASL overexpression, followed by treatment with 40 μM OXA for 48 h. The values indicate the mean ± standard deviation (SD) of three independent experiments. Statistical significance is indicated as follows: “ns” indicates no statistically significant difference, “*” indicates *P* < 0.05, “**“ indicates *P* < 0.05, and “^▲^” indicates *P* < 0.001.

To further clarify the relationship between OXA and OASL, we treated AGS, MKN 45, and HGC27 cells with different concentrations (0, 10, 20, and 40 μM) for 48 h, and OASL protein expression was detected by western blot. The results showed that OASL protein expression increased dose-dependently compared to the control group (*P* < 0.001; Fig. 3A). RT-PCR analysis revealed significant upregulation of OASL mRNA levels in response to OXA exposure(*P* < 0.05; Fig. 3B). These results indicate that both protein and mRNA expression of OASL are positively correlated with OXA concentration.

We next investigated whether OASL modulates GC cell sensitivity to OXA. AGS and MKN45 cells were knocked down with OASLand treated with OXA at different concentrations (0, 10, 20, and 40 μM) for 48 h, and the rate of increase of apoptosis was determined using flow cytometry (Annexin-FITC/PI double staining). The results showed that in AGS and MKN45 cells, the apoptosis rate in the si-OASL group compared with that in the control group was increased (*P* < 0.05; Fig. 3C; Supplementary Fig. S3). Consistently, LDH release assays confirmed enhanced cytotoxicity in OASL-knockdown groups (*P* < 0.05; Fig. 3E). Similarly, we treated HGC27 cells with OASL and overexpressed it with 40 μM OXA for 48 hours. Apoptosis and LDH levels in the 40 μM OXA + OASL group were significantly lower than those in the control group (*P* < 0.05; Fig. 3D, E). Combined with the above results, it was concluded that OASL inhibits OXA-induced apoptosis and reduces chemosensitivity in GC cells.

#### Effect of OASL on oxaliplatin-induced immunogenic cell death of gastric cancer cells

**Fig. 4:**
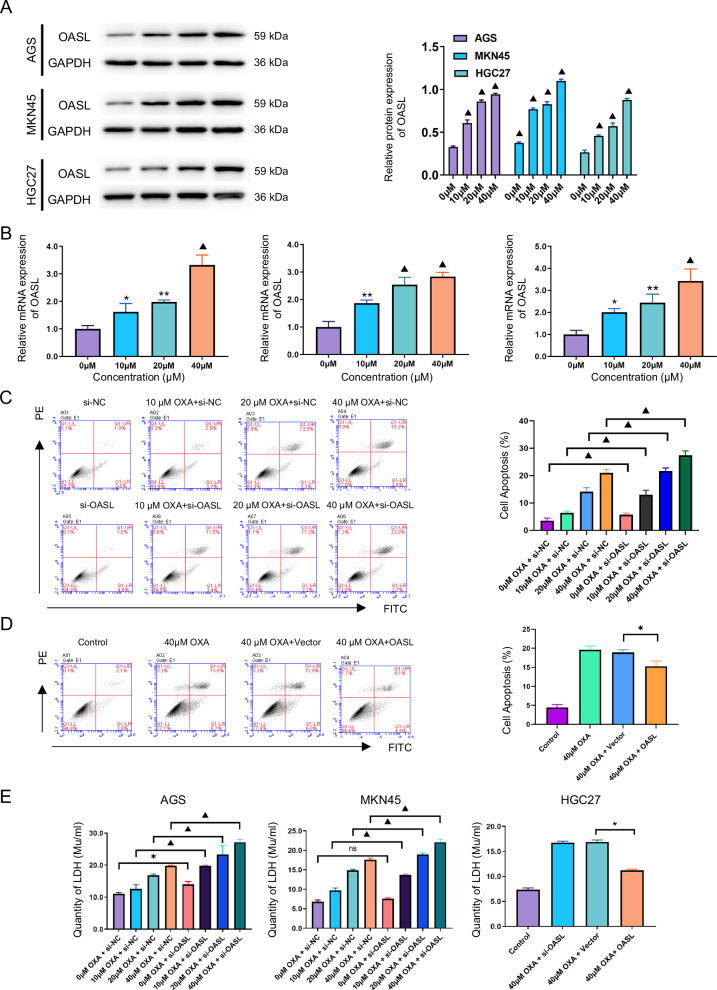
OASL can reduce OXA-induced immunogenic cell death in GC cells. **A** AGS cells were treated with 40 μM OXA combined with si-OASL for 48 h, and changes in CRT were observed using immunofluorescence. **B** AGS cells were treated with 40 μM OXA combined with si-OASL for 48 h, the expression level of CRT on cell membrane was measured using flow cytometry with dead cells, and a statistical chart was generated. **C** AGS cells were treated with 40 μM OXA combined with si-OASL for 48 hours, and the statistical plot of HMGB1 content in cell supernatant of cell culture medium was detected using ELISA assay. **D** AGS cells were treated with 40 μM OXA combined with si-OASL for 48 h, and statistical plot of ATP content in cell supernatant of cell culture medium was detected using ATP kit. **E** AGS and MKN45 cells were treated with 40 μM OXA in combination with si-OASL for 48 h, and HGC27 cells were treated with 40 μM OXA in combination with overexpression of OASL for 48 hours. The expression levels of HSP70 and HSP90 proteins in AGS, MKN45, and HGC27 cells were determined by Western blot, and statistical graphs were obtained. The values indicate the mean ± standard deviation (SD) of three independent experiments.Scale bar: 100 μm. Statistical significance is indicated as follows: “*“ indicates *P* < 0.05, “**“ indicates *P* < 0.01, and “***“ indicates *P* < 0.001.

To further investigate the role of OASL in OXA-induced ICD in GC cells, AGS and MKN45 cells were treated with 40 μM OXA combined with si-OASL for 48 h; HGC27 cells were treated with 40 μM OXA in combination with overexpression of OASL for 48 h, and the expression levels of OASL were determined by western blot. Western blot analysis confirmed that OXA treatment significantly induced OASL expression in both models(*P* < 0.01; Supplementary Fig. S4). We further examined the effect of OASL on ICD-associated DAMPs in OXA-induced GC cells in AGS and MKN45 cells. The experiments were divided into control, OXA, OXA+si-NC, and OXA+si-OASL groups with an OXA concentration of 40 μM, and ICD-associated DAMPs were detected after 48 h in AGS cells. The experimental results showed that the OXA+si-OASL group-related DAMPs (CRT agglomeration on the membrane surface, the content of HMGB1 and ATP in the medium supernatant, and HSP70 and HSP90 protein expression in cells) were significantly higher than those in the OXA+si-NC group (*P* < 0.05; Fig. 4A–E). The same method was used for MKN45 cells (*P* < 0.05; Supplementary Fig. S5). Combined with the above results, we concluded that OASL knockdown enhances OXA-induced ICD in GC cells.

To further validate the above results, we treated OASL overexpression combined with 40 μM OXA in HGC27 cells for 48 hours, grouped them, and detected them as described above. The experimental results showed that the OXA + OASL group showed significantly less ICD related DAMPs (*P* < 0.05; Supplementary Fig. S6). Combined with the above results, it was concluded that the overexpression of OASL inhibited OXA-induced ICD in GC cells.

In conclusion, these findings demonstrate that OASL acts as a negative regulator of OXA-induced ICD in GC cells

### OASL regulation of the cGAS-STING signaling pathway inhibits oxaliplatin-induced immunogenic cell death

Our prior data demonstrate that OASL promotes GC cell proliferation, invasion, and migration while suppressing ICD, consequently diminishing OXA chemosensitivity. Nevertheless, the precise molecular mechanism underlying these effects remains uncharacterized. This section therefore, investigates the following key aspects:

#### mRNA sequencing predicts the potential downstream signaling pathways of OASL

**Fig. 5:**
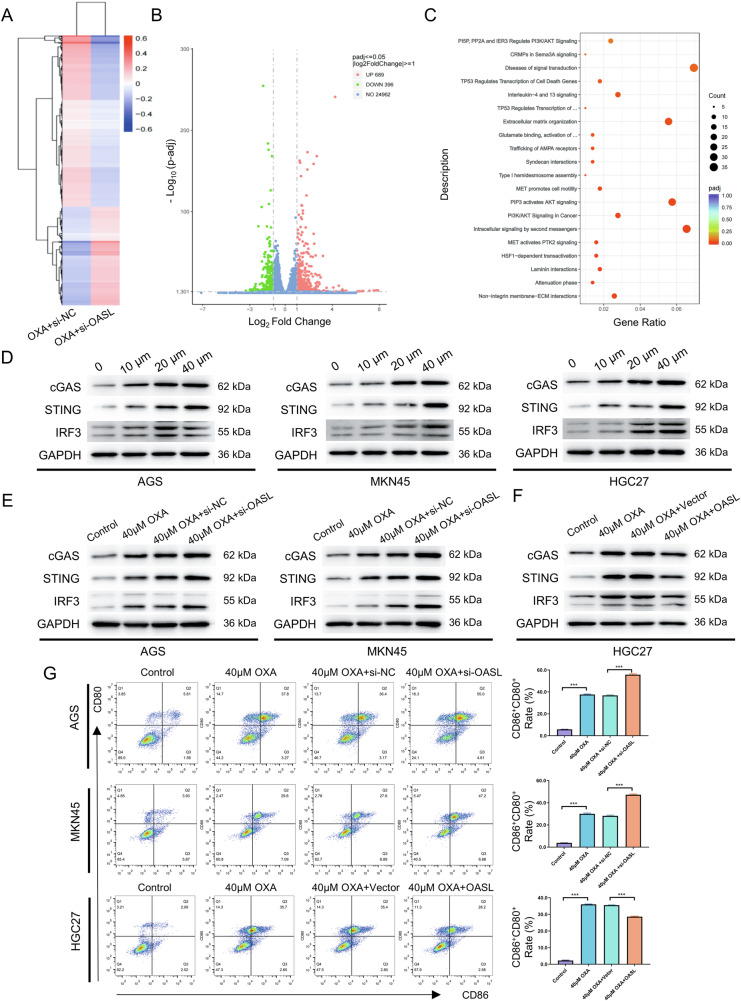
OASL regulates the expression of key proteins in the cGAS-STING signaling pathway. **A** All the detected mRNA expression levels were visualized using the heatmap (*n* = 3). **B** The Intervention up-regulated and down-regulated mRNA were displayed in groups using a volcano map. **C** Reactome enrichment analysis of the differential mRNA. Reactome enrichmentanalysis of the differentially expressed genes showed significant enrichment in intracellular signaling by second messenger, The cGAS recognizes and binds DNA in the cytoplasm, catalyzes the synthesis of ATP and GTP into cGAMP; cGAMP acts as a second messenger to bind and activate STING. **D** AGS, MKN45, and HGC27 cells were treated with OXA at various concentrations (0, 10, 20, and 40 μM) for 48 h, and the expression levels of key proteins of the cGAS-STING signaling pathway were determined by Western blot. **E** AGS and MKN45 cells were treated with 40 μM OXA combined with si-OASL for 48 h, **F** HGC27 cells were treated with 40 μM OXA in combination with overexpression of OASL for 48 h, and the expression levels of key proteins of the cGAS-STING signaling pathway were determined by Western blot. **G** AGS and MKN45 cells were treated with 40 μM OXA combined with si-OASL for 48 h, HGC27 cells were treated with 40 μM OXA in combination with overexpression of OASL for 48 h, and then co-culture with immature DC cells, and the expression level of CD86/CD80 on the surface of DC cells were detected using by flow cytometry, and statistical graphs were obtained. The values indicate the mean ± standard deviation (SD) of three independent experiments. Statistical significance is indicated as follows: “***“ indicates *P* < 0.001.

To investigate how OASL suppresses ICD in GC cells, mRNA sequencing of MKN45 cells (OXA+si-NC vs. OXA+si-OASL) revealed differential transcriptomes visualized by heatmap (Fig. 5A). Volcano plot analysis identified 1243 differentially expressed genes (Fig. 5B). Reactome enrichment demonstrated significant cGAMP second messenger pathway involvement (Fig. 5C), which centers on cGAS-mediated cGAMP synthesis and subsequent STING-dependent immune activation. These findings indicate OASL regulates ICD through the cGAS-STING signaling pathway.

#### OASL regulates the expression of key proteins of the cGAS-STING signaling pathway

To further explore the relationship between OASL and cGAS-STING pathway, we first explored the correlation between OXA and this pathway. AGS, MKN45, and HGC27 cells were treated with OXA at various concentrations (0, 10, 20, and 40 μM) for 48 h, and western blot was performed to detect the expression of key proteins of the cGAS-STING signaling pathway. Results showed that the protein expression of cGAS, STING, IRF3, P-STING, P-TBK1, and P-IRF3 increased at all tested concentrations relative to the control (0 μM) group (*P* < 0.05; Fig. 5D; Supplementary Fig. S7). OXA treatment dose-dependently enhanced cGAS-STING signaling pathway activation in GC cells, significantly upregulating expression of cGAS, STING, IRF3, P-STING, P-TBK1, and P-IRF3.

Next, the relationship between OASL and cGAS-STING was further analyzed to test the effect of OASL knockdown and overexpression on the protein expression levels of cGAS, STING, RIF3, P-STING, P-TBK1, and P-IRF3 in GC cells. The experiments were divided into control, OXA, OXA+si-NC, and OXA+si-OASL groups, and 40 μM OXA was applied to AGS and MKN45 cells for 48 hours, respectively, and the expression of key proteins of the cGAS-STING signaling pathway was determined by western blot. The results showed that the protein expression of cGAS, STING, IRF3, STING, P-TBK1, and P-IRF3 in the OXA+si-OASL group increased (*P* < 0.05; Fig. 5E; Supplementary Fig. S7). HGC27 cells were treated with OASL combined with 40 μM OXA for 48 hours and grouped and tested as described above. The results showed that the protein expression of cGAS, STING, IRF3, STING, P-TBK1, and P-IRF3 were decreased in the OXA + OASL group than in the OXA+Vector group (*P* < 0.05; Fig. 5F; Supplementary Fig. S7).

The experiments were divided into control group, OXA group, OXA+si-NC group, and OXA+si-OASL group, and 40 μM OXA was applied to AGS and MKN45 cells for 48 h, and then co-culture with immature DC cells for 24 h, compared to OXA+si-NC group, the expression level of CD86/CD80 on the surface of DC cells was increased in OXA+si-OASL group (*P* < 0.001, Fig. 5G). HGC27 cells were treated with OASL combined with 40 μM OXA for 48 h and grouped and tested as described above, compared to OXA+Vector group, the expression level of CD86/CD80 on the surface of DC cells was decreased in OXA + OASL group (*P* < 0.001, Fig. 5G).

Based on these results, OASL can regulate the cGAS-STING pathway and exert biological effects.

#### OASL critically depends on the cGAS-STING signaling pathway to mediate oxaliplatin-induced immunogenic cell death

**Fig. 6:**
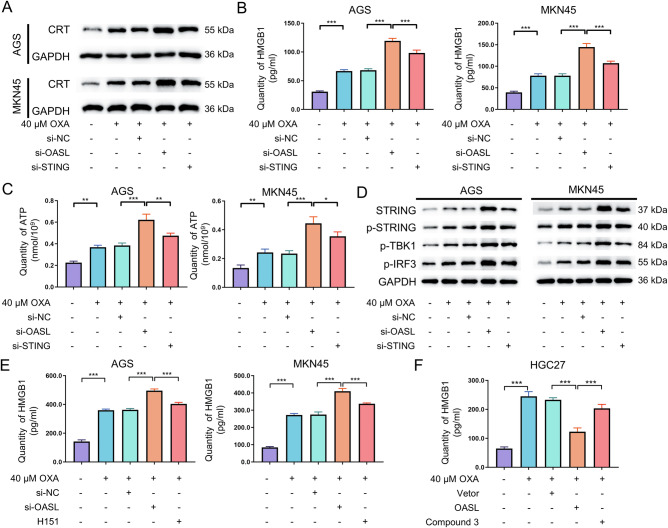
OASL-dependent cGAS-STING signaling pathway to regulate OXA-induced immunogenic cell death. **A** AGS and MKN 45 cells were treated with 40 μM OXA in combination with si-OASL and si-STING for 48 h, the expression levels of CRT protein in AGS and MKN45 cells were determined by Western blot. **B** AGS and MKN45 cells were treated with 40 μM OXA in combination with si-OASL and si-STING for 48 h, the statistical plots of HMGB1 content in cell supernatants were detected using ELISA assay. **C** AGS and MKN45 cells were treated with 40 μM OXA in combination with si-OASL and si-STING for 48 hours, the statistical plots of ATP content in cell supernatant detected using ATP kit. **D** AGS and MKN 45 cells were treated with 40 μM OXA in combination with si-OASL and si-STING for 48 h, and the expression levels of key proteins of the cGAS-STING signaling pathway were determined by Western blot. **E** AGS and MKN 45 cells were treated with 40 μM OXA in combination with si-OASL and supplemented with H151(an inhibitor of the cGAS-STING signaling pathway) for 48 h, the statistical plots of HMGB1 content in cell supernatants of cell culture medium were detected using ELISA assay. **F** HGC27 cells were treated with 40 μM OXA combined with overexpressed OASL and supplemented with Compound 3 (an activator of the cGAS-STING signaling pathway) for 48 h, statistical plot of ATP content in cell supernatant of cell culture medium was detected using ATP kit. The values indicate the mean ± standard deviation (SD) of three independent experiments. Statistical significance is indicated as follows: “*“ indicates *P* < 0.05, “**“ indicates *P* < 0.01, and “***“ indicates *P* < 0.001.

To determine whether OASL inhibits OXA-induced ICD by regulating the cGAS-STING signaling pathway, we designed a rescue experiment. The experiments were divided into control group, OXA group, OXA+si-NC group, OXA+si-OASL group, and OXA+si-OASL+si-STING group, and the detection of OXA concentration was 40 μM, followed by AGS and MKN 45 cells. In the AGS and MKN45 cells, compared with the OXA+si-OASL group, the expression level of CRT protein were decreased (*P* < 0.001; Fig. 6A) and the contents of HMGB1 and ATP in the cell supernatant were reduced(*P* < 0.05; Fig. 6B, C) and the expression levels of STING, P-STING, P-TBK1, and P-IRF3 were decreased in OXA+si-OASL+ si-STING group(*P* < 0.05; Fig. 6D).

The experiments were divided into control group, OXA group, OXA + si-NC group, OXA+si-OASL group, OXA + OASL group, and OXA+si-OASL+inhibitor group, and the detection of OXA concentration was 40 μM, followed by AGS and MKN 45 cells (only key DAMPs such as HMGB 1 and HSP70 and HSP90 were detected in this section). In the AGS and MKN 45 cells, compared with the OXA + si-OASL group, after adding the cGAS-STING signaling pathway inhibitor H151, found that the contents of HMGB1 in the cell supernatant was reduced, the expression levels of HSP70 and HSP90 were decreased (*P* < 0.001; Fig. 6E; Supplementary Fig. S8). In HGC27 cells, compared with the OXA + OASL group, after adding the cGAS-STING signaling pathway activator Compound 3, found that the contents of HMGB1 in the cell supernatant was increased, the expression levels of HSP70 and HSP90 were increased (*P* < 0.001; Fig. 6F; Supplementary Fig. S8).

Based on the above findings, OASL may rely on the cGAS-STING signaling pathway to regulate OXA-induced ICD.

#### OASL binds to cGAS proteins to inhibit the cGAS-STING signaling pathway

**Fig. 7:**
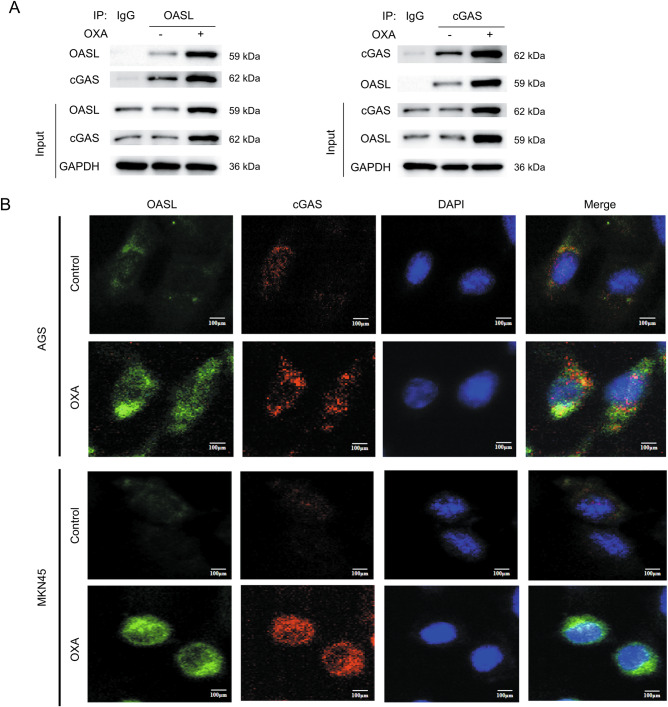
OASL binds to cGAS proteins to inhibit the cGAS-STING signaling pathway. **A** Co-IP results plots for OASL and cGAS(*n* = 3). **B** Immunofluorescence plots verifying the binding of OASL and cGA by adding OXA treatment to AGS and MKN cells (*n* = 3). Scale bar: 100 μm.

Based on these findings, we further investigated the mechanism through which OASL modulates the cGAS-STING signaling pathway. The association between OASL and cGAS proteins was first revealed in co-immunoprecipitation (Co-IP) assays (Fig. 7A). To further clarify this interaction, we treated AGS and MKN45 cells with OXA. Immunofluorescence analysis showed increased expression and direct colocalization of OASL and cGAS in OXA-treated cells compared to controls (Fig. 7B).

#### Mouse tumor-bearing model verified that knockdown of OASL can inhibit oxaliplatin-induced immunogenic cell death in gastric cancer cells

**Fig. 8:**
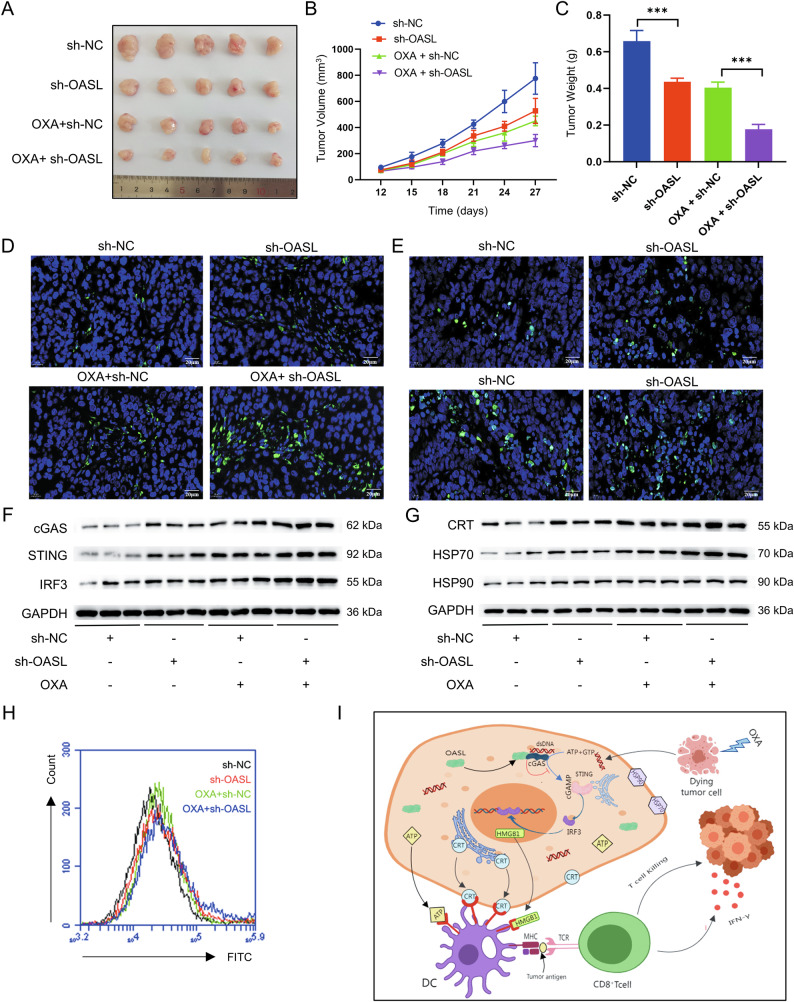
A mouse tumor-bearing model verified that knockdown of OASL improved OXA-induced ICD in GC cells. **A** In vivo tumorigenesis of MFC cells with sh-OASL in mice. **B** Vernier calipers measured the length and width of subcutaneous tumor-bearing tissue of inoculated mice from 12 days after inoculation, and plot the growth curve of tumor-bearing tissue. **C** Weight statistical plot of the tumors. **D** Apoptosis and statistics of mouse tumor-bearing tumors detected by TUNEL staining. **E** CD8 expression and statistics of mouse tumor bearing by immunohistochemical fluorescence staining. **F** Expression and statistical plots of key proteins of cGAS-STING signaling pathway determined by Western blot. **G** Expression and statistical plots of the CRT and HSP70, and HSP90 proteins associated with ICD were determined by Western blot. **H** The expression level of CRT on the cell membrane was measured using flow cytometry with dead cells. **I** The simulation diagram illustrating the mechanism how OASL exerts its biological effects. The values indicate the mean ± standard deviation (SD) of three independent experiments.Scale bar: 20 μm. Statistical significance is indicated as follows: “***“ indicates *P* < 0.001.

The above in vitro experiments demonstrated that OASL inhibits cGAS-STING signaling pathway to reduce ICD in GC cells. To further verify the mechanism of OASL action in GC cells, we conducted in vivo experiments. C57/BL6 mice were subcut-aneously vaccinated with OASL shRNA knockdown MFC cells and NC-transfected MFC cells, and the experiments were divided into four groups: sh-NC, sh-OASL, OXA+sh-NC, and OXA+sh-OASL. Tumor volume decreased in the sh-OASL group compared to the sh-NC group, and the OXA+sh-OASL group exhibited a statistically significant reduction (*P* < 0.05; Fig. 8A, B). The weight of the tumor-bearing tissue was significantly lower in the sh-OASL group than that in the sh-NC + OASL group (*P* < 0.01; Fig. 8C). The weight of tumor-bearing tissue was significantly lower in the sh-OASL group than that in the sh-NC OASL group. TUNEL staining is a highly sensitive method for detecting apoptosis. The apoptosis rate was calculated by counting positive and negative cells, which showed significantly more TUNEL-positive cells and higher apoptosis rates in the sh-OASL group than in the sh-NC group, and in the OXA + sh-OASL group than in the OXA+ sh-NC group. (*P* < 0.01; Fig. 8D). To further define CD8^+^ T-cell infiltration in tumor-infiltrating lymphocytes (TILs), we performed immunofluorescence staining for CD8. Results showed significantly increased CD8^+^ T cells in the sh-OASL group compared with the sh-NC group, and in the OXA+sh-OASL group compared with the OXA+sh-NC group. (*P* < 0.01; Fig. 8E).

To further clarify the effect of OASL on the cGAS-STING signaling pathway, we examined the expression of key proteins of the cGAS-STING signaling pathway by western blot, and the results showed that compared with the sh-NC group, the protein expression levels of cGAS, STING, and IRF3 were significantly higher in the sh-OASL group than in the OXA+sh-NC group, and the protein expression levels of cGAS, STING, and IRF 3 were significantly higher in the OXA+sh-OASL group (*P* < 0.001; Fig. 8F).

To further clarify the effect of OASL on ICD, we detected the expression of ICD-related proteins by western blot. Results showed increased protein expression of CRT and HSP70 and HSP90 in the sh-OASL group compared with the sh-NC group, and in the OXA + sh-OASL group compared with the OXA + sh-NC group. Flow cytometry independently confirmed elevated CRT surface exposure. (*P* < 0.05; Fig. 8G).

In conclusion, the mouse tumor-bearing model verified that knockdown of OASL enhanced the activation of the cGAS-STING signaling pathway and enhanced OXA-induced ICD in GC cells,thus increasing the sensitivity of OXAchemotherapy.

## Discussion

GC is a common malignant tumor of the digestive tract that seriously affects human health. At present, GC treatment is mainly surgery, supplemented by radiotherapy and chemotherapy, in addition to targeted and immune comprehensive therapy. Currently, the CSCO guidelines recommend first-line treatment for advanced GC, with chemotherapy as the backbone, which can be combined with immunotherapy or targeted therapy based on specific clinical conditions. Therefore, chemotherapy is indispensable for treating advanced GC [7]. OXA is one of the most commonly used platinum chemotherapy drugs. Its mechanism of action is the complexation of platinum atoms with DNA, which inhibits the replication and transcription of tumor cells. However, in recent years, OXA has been found in clinical medications, and this process is often easy, leading to serious drug resistance [36]. Although chemotherapy can effectively retreat or temporarily eliminate tumors in the short term, long-term use of chemotherapeutic drugs in patients often leads to MDR, which is one of the main causes of treatment failure in GC patients [37, 38]. Therefore, reversing OXA resistance and improving chemotherapy sensitivity remain a challenge.

Innovatively starting from antitumor immunological mechanisms, we proposed that OASL may critically regulate OXA chemosensitivity. To verify the specific regulatory mechanism, we first determined whether OXA could induce apoptosis in GC cells in vitro. Our results showed that OXA inhibited proliferation and promoted apoptosis in GC cells. We further examined whether ICD was induced during OXA-mediated apoptosis. ICD generation depends critically on DAMPs, including CRT surface exposure, HMGB1 release, and ATP secretion. These molecules activate and recruit antigen-presenting cells, leading to effector T cell activation and subsequent antitumor immune responses [39]. The current study found that the immune response has a direct relationship with three DAMPs, including CRT, HMGB1, and ATP, and HSP70 and HSP90 acts as molecular chaperones [40–42]. Bortezomib and anthracyclines can induce exposure of HSP70 and HSP90 to the cell surface and act as carriers for antigenic peptides [43]. Our results confirmed that OXA dose-dependently increased HSP70 and HSP90 protein expression in GC cells. Thus, OXA induces ICD in GC cells in a dose-dependent manner. We subsequently modulated OASL expression and treated cells with OXA to quantify DAMP expression and release. The results showed that knockdown of OASL enhanced OXA-induced ICD in GC cells, whereas overexpression of OASL inhibited OXA-induced ICD in GC cells. In conclusion, OASL inhibits OXA-induced ICD in GC cells. These findings confirm that OASL critically maintains chemosensitivity in GC.

To explore the molecular mechanism by which OASL reduces OXA-induced ICD in GC cells, we first performed mRNA sequencing of MKN45 cells (si-NC + OXA and si-OASL + OXA groups). Through differential expression and reactome enrichment analysis, the results showed significant enrichment in the second messenger signaling pathway. In 2011, Russell E. Vance’s team found that STING itself directly senses c-di-GMP DNA sensor in animal cells [44]. Along with the research, it was not proposed until 2013 that cGAMP could directly activate STING as a second messenger, and cGAS was a key synthetic enzyme upstream of cGAMP [45]. The composition and activation mechanism of the cGAS-STING signaling pathway are now clearly defined: cytosolic dsDNA binds cGAS, which catalyzes cGAMP synthesis from ATP and GTP. cGAMP then activates STING, triggering its translocation to the Golgi apparatus, where it forms the TBK1-STING-IRF3 complex to induce type I interferon production [46, 47]. The cGAS-STING signaling pathway is the major pathway responsible for identifying the immune response to cytosolic DNA [48]. With the current research, the cGAS-STING signaling pathway is also playing an increasingly important role in anti-tumor immunity. Based on the above, we hypothesized that OASL exerts its biological effects through cGAS-STING signaling pathway.

To delineate the OASL-cGAS-STING functional relationship, we first established that OXA dose-dependently upregulated cGAS, STING, IRF3, P-STING, P-TBK1, and P-IRF3 in GC cells. Crucially, OASL knockdown enhanced OXA-induced activation of this pathway, while OASL overexpression suppressed it, confirming OASL’s regulatory role. Rescue experiments demonstrated OASL governs OXA-induced ICD through cGAS-STING signaling pathway: (1) STING inhibitor H151 reduced HMGB1 release and HSP70 and HSP90 expression versus OXA+si-OASL(*P* < 0.05); (2) cGAS-STING activator Compound 3 elevated these ICD markers compared to OXA + OASL(*P* < 0.05); and most critically, (3) dual OASL/STING knockdown (OXA+si-OASL+si-STING) reversed ICD hallmarks-significantly decreasing CRT exposure, ATP/HMGB1 release, and STING/TBK1/IRF3 phosphorylation versus OXA+si-OASL (*P* < 0.05). These data conclusively demonstrate OASL modulates oxaliplatin chemosensitivity in GC by regulating ICD via the cGAS-STING signaling pathway. Based on the above results, we further explored the mechanism of OASL regulating cGAS, we confirmed the association between OASL and cGAS proteins by Co-IP and immunofluorescence, which is consistent with the report: where OASL binds to the DNA sensor cGAS during DNA viral infection, thereby inhibiting IFN induction and enhancing DNA viral replication [49].

A growing number of studies have confirmed that the infiltration of tumor lymphocytes (tumor-invasive lymphocytes, TILs) is closely related to the overall survival of GC patients, and that high levels of tumor lymphocytes can significantly reduce the risk of recurrence and mortality in patients [50–52]. As the major component of TILs, CD8^+^TIL are considered to be one of the main drivers of anti-tumor immunity [53]. In the immune checkpoint treatment of GC, CD8^+^ infiltration of TIL can be used as a potential biomarker for prognosis prediction. In vivo, OASL knockdown synergized with OXA treatment to activate cGAS-STING signaling pathway and enhance ICD. This combination significantly promoted CD8^+^T-cell infiltration within TILs. The augmented TIL infiltration correlated directly with OXA-induced ICD enhancement. Critically, OXA effectively induced ICD in tumor-bearing mice, and OASL knockdown further potentiated this effect.

While established mechanisms of OXA resistance in gastric cancer involve JUNB-mediated MAPK hyperactivation, EphA2-induced EMT, METTL3-dependent DNA repair/stemness maintenance, and LINC00641-regulated autophagy, our study reveals a distinct immunosuppressive axis mediated by OASL. Unlike these tumor-intrinsic adaptations, we demonstrate that OASL impairs ICD by directly binding cGAS and suppressing cGAS-STING signaling pathway, thereby inhibiting dendritic cell maturation and CD8^+^T-cell infiltration. This contrasts with epigenetic or autophagic pathways that primarily enhance cellular survival, positioning OASL as a unique resistance factor operating through immune evasion. Critically, OASL knockdown synergized with oxaliplatin to restore ICD in vivo, suggesting that targeting this pathway could complement existing strategies against classical resistance mechanisms.

In conclusion, OASL critically inhibits cGAS-STING signaling pathway to suppress OXA-induced ICD in GC cells, thereby decreasing chemosensitivity to OXA.

## Materials and methods

### Cell culture

GC cell lines (AGS, MKN45, and HGC27) were purchased from Cobioer (Nanjing, China). AGS, MKN45, and HGC27 cells were cultured in RPMI 1640 (BasalMedia, Shanghai, China) containing 10% fetal bovine serum (FBS) in a humidified incubator with 5% CO_2_ at 37 °C.

The cells were incubated with oxaliplatin(OXA) (S1224, Selleck, China), STING inhibitor H151(S6652, Selleck, China),diABZI STING agonist(Compound 3) (S8796, Selleck, China).

### Cell transfection

siRNA (si-OASL:CCATCACGGTCACCATTGT and si-STING: GGUCAUAUUAC AUCGGAUA) and the negative control (si-NC) were purchased from RiboBio (Guangzhou, China). OASL-amplified products were ligated into the pc-DNA3.1 expression vector was purchased from Tsingke Biotechnology (Beijing, China). si-OASL and si-NC were transfected into AGS and MKN45 cells, respectively, using Lipofectamine 2000 (Invitrogen, USA). The empty vector (Vector) and pcDNA3.1-OASL were transfected into HGC27 cells using Lipofectamine 2000. After 48 h, the transfection efficiency was measured by real-time quantitative polymerase chain reaction (RT-qPCR) and western blot.

### Cell counting kit-8 (CCK-8)

AGS, MKN45, and HGC27 cells were seeded in 96 well plates (5 × 10^3^ cells/well). After incubation at the specified time, the CCK-8 solution (Beyotime, Shanghai, China) was added and incubated for another 2 h. The OD450 values were measured using a microplate reader (Bio-Rad, USA).

### Flow cytometry

AGS, MKN45, and HGC27 cells were washed with PBS and digested with trypsin (0.25%). The cells were collected after centrifugation at 1000 g for 5 min, suspended in PBS, and counted. Subsequently, the cells (2 × 10^5^ cells/well) were centrifuged and resuspended in 195 μL of Annexin V-FITC binding solution. Annexin V-FITC (5 μL) and propidium iodide (PI) staining solution (10 μL) were added and mixed gently. After incubation in the dark for 20 min at 37 °C, apoptosis was assessed by flow cytometry.

### Flow cytometric analysis of cell surface CRT

Seed AGS, MKN45, and HGC27 cells in 6-well plates. Following experimental treatments, harvest cells by centrifugation at 1000 rpm for 5 min. Wash cell pellets twice with ice-cold PBS using identical centrifugation parameters. Incubate with diluted anti-CRT primary antibody at 4 °C for 60 min under light-protected conditions. Perform two PBS washes to remove unbound primary antibody. Add fluorophore-conjugated secondary antibody and incubate at room temperature for 30 min protected from light. Complete two final PBS washes and resuspend cells in 300–500 μL PBS. Acquire data using flow cytometry.

### DC coculture maturation assay

Human dendritic cells (DCs) were generated from peripheral blood mononuclear cells isolated via density gradient centrifugation. PBMCs were cultured for 5 days in RPMI-1640/10% FBS supplemented with GM-CSF (50 ng/mL) and IL-4 (20 ng/mL). Conditioned medium was collected from AGS, MKN45, and HGC27 cells (transfected with si-OASL/OASL and treated with 40 μM OXA for 24 h) after centrifugation at 3000 rpm for 5 min. For co-culture, DCs were exposed to 50% conditioned medium for 24 h with 5% CO2 at 37 °C. DC maturation was assessed by flow cytometric analysis of CD86/CD80 surface expression.

### Western blot

Total protein was extracted from the tissues and cells using RIPA lysis buffer (Beyotime, Shanghai, China), and the protein concentration was detected using the BCA Protein Assay kit (Beyotime, Shanghai, China). Protein samples (30 μg) were separated using 10% SDS-PAGE and transferred onto PVDF membranes. The membranes were incubated with primary. The antibodies were incubated overnight at 4 °C. Thereafter, the membranes were incubated with HRP-conjugated secondary antibody (ab205718, Abcam, USA) for 2 h at room temperature. Subsequently, the bands were visualized using an ECL kit (Beyotime, Shanghai, China) and quantified using Image J software. The following primary antibodies were used for the western blot analysis: OASL(ab229136, Abcam, UK), cGAS (ab224144, Abcam, UK), STING (ab252560, Abcam,UK), IRF3 (ab245341, Abcam, UK), CRT (ab227444, Abcam, UK), HSP90 (ab203126, Abcam, UK), HSP70 (ab194360, Abcam, UK), CD8 (ab316778, Abcam, UK), GAPDH (10494-1-AP, Proteintech, USA), P-STING (AP1369, ABclonal, CN), P-TBK1 (AP1026, ABclonal, CN), P-IRF3 (AP0995, ABclonal, CN).

### Co-IP

Cells were collected 48 h after transfection and gently rinsed twice with precooled 1 × PBS. Freshly prepared cracking working solution was added, cracked on ice for 30 min, transferred to an EP tube, and sonicated on ice for 3 minutes. The supernatant was centrifuged and transferred to a new EP tube. Protein A/G PLUS Agarose (15 μL) (Beyotime, Shanghai, China) was added to each tube and incubated at 4 °C for 1 h. The supernatant was centrifuged and transferred to a new EP tube. Lysis buffer (60 μL)was added to 15 μL of 5 × loading buffer and boiled at 1000 W for 10 min. Antibody (1.5 μg/tube) was added to the remaining lysate and incubated at 4 °C in the suspension apparatus for 1 h. Protein A/G PLUS agarose (40 μL/tube) was added to a mixture of antibody and lysis buffer and incubated overnight at 4 °C in a suspension apparatus. The beads were washed four times with lysis solution. After suction, 40 μL of lysis buffer was added to each tube, 40 μL of 2 × loading buffer was added, and the sample was boiled at 4 °C for 10 min. Western blot analysis and detection were performed.

### ELISA

The cell suspensions of each group were centrifuged, the cell supernatant was collected, and the instructions of the corresponding ELISA kit (Beyotime, Shanghai, China) were followed to sequentially detect the levels of HMGB1 in the supernatant of the group cells.

### Quantification of extracellular ATP release

The cell supernatant was collected after processing, and an ATP colorimetric detection kit (Beyotime, Shanghai, China) was used to measure ATP levels in the supernatant of the group cells.

### Measurement of Lactate Dehydrogenase(LDH) release

The cell supernatant was collected after processing the cells, and an LDH assay kit (Beyotime, Shanghai, China) was used to detect the LDH levels in the supernatant of the group cells.

### Immunofluorescence

Take cells treated with each group, fix them with 4% paraformaldehyde for 10 minutes, wash them with PBS, and then use 0.5% Triton X-100 was passaged at room temperature for 20 min, then blocked and added with primary antibody. The cells were incubated at room temperature for 1 h, followed by the addition of labeled secondary antibodies. Cells were counterstained with DAPI, observed under a fluorescence microscope, and photographed.

### Mouse tumor-bearing mode

C57/BL6 mice (*n* = 20, male, 6–8 weeks old) were purchased from Jinan Pengyue Experimental Animal Breeding Co. Ltd. SPF mouse-specific feed and high-pressure sterilized water were used for feeding in the experimental animal center.

1. Mice were kept in a relatively sterile constant-temperature environment for one week and randomly divided into four groups: sh-NC (*n* = 5), sh-OASL (*n* = 5), OXA + sh-NC (*n* = 5), and OXA + sh-OASL (*n* = 5). shRNA (sh-OASL:GAGTGTGACTA ACAGAGTACC) was purchased from RiboBio(Guangzhou, China).
2. The transfected MFC cells were adjusted for cell density to 1.5 × 10^6^/mL, and a 0.2 mL cell suspension was seeded in the right armpit of mice. A tumor diameter of 0.5 cm, it indicated successful molding.
3. The dose was calculated using a human-to-mouse surface area ratio of 0.002:6, and OXA was 10 mg/kg was 0.2 mL (1 time/2 d).
4. Tumor volume was measured every three days after injection (volume =length and width×2/2). The mouse was killed 15 days later, and the tumor bodies were dissected, weighed, and photographed.
5. The tumor tissue was divided into three parts, with a portion of fresh tissue extracted for protein for western blot, another portion of fresh tissue subjected to TUNEL staining, and the last part of fresh tissue dehydrated and subjected to CD8 immunohistochemical staining.

### Statistical analysis

GraphPadPrism17 was used for statistical analyses, and data are expressed as mean ± standard deviation (SD). The Student’s t-test was used to analyze the differences between the two groups. *P* < 0.05 was considered statistically significant. Three replicates were used for each experiment.

## Supplementary information

Supplementary

WB

## Data Availability

The data that support the findings of this study are available on request from the corresponding author. The data are not publicly available due to privacy or ethical restrictions.
