# Supplementary material for: Effect of OASL on oxaliplatin-induced immunogenic cell death in gastric cancer via the cGAS-STING signaling pathway
Source: Cell Death Discov. 2025 Nov 21;12:20. doi: 10.1038/s41420-025-02850-w (PMC12804755; doi:10.1038/s41420-025-02850-w)
Supplement: Supplementary file 1 — Supplementary [file 41420_2025_2850_MOESM1_ESM.pdf]

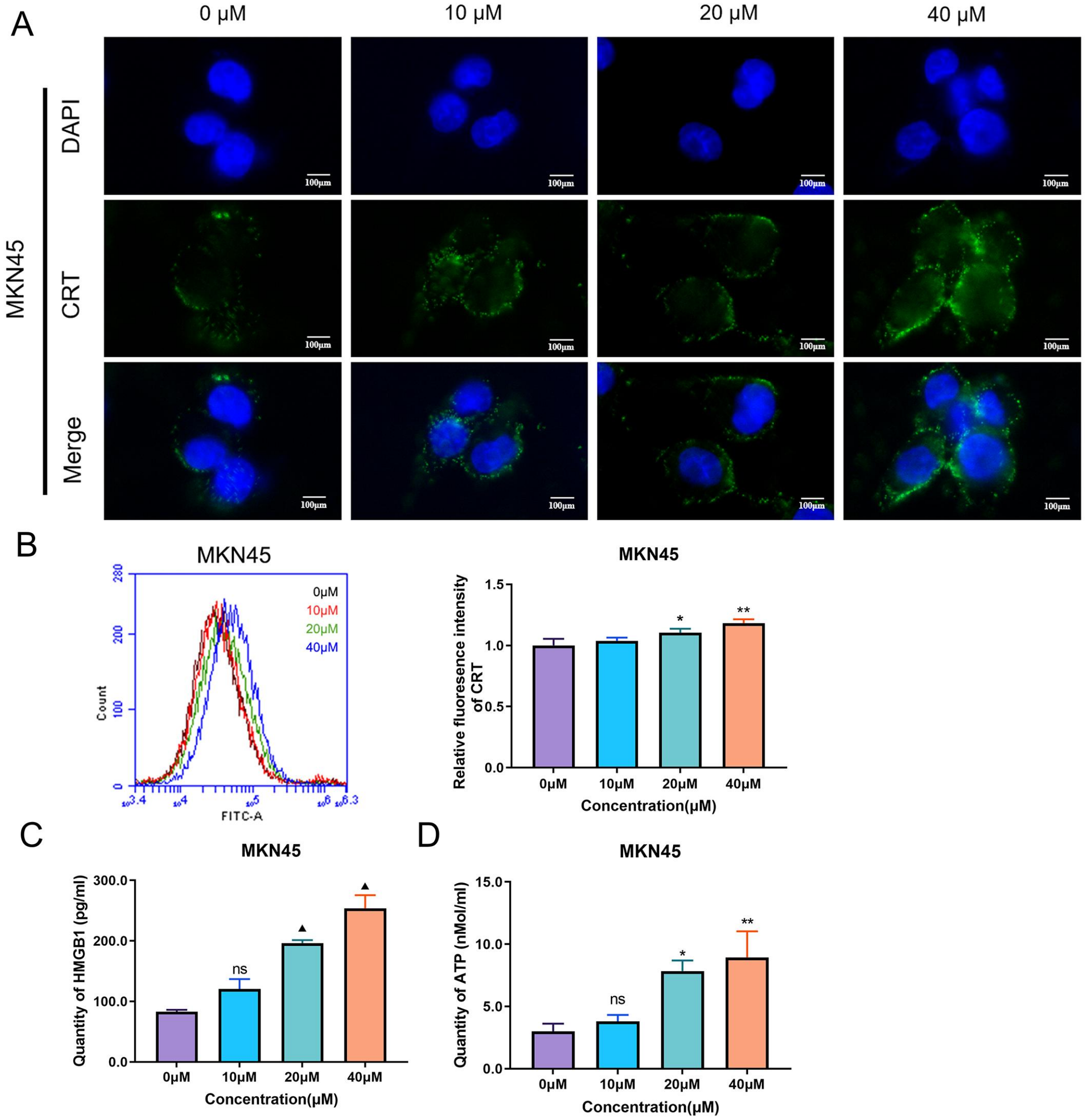

Supplementary Figure S1 OXA was able to induce immunogenic cell death in MKN45 cells (A) MKN45 cells were treated with OXA at various concentrations (0,10,20 and 40 μM) for 48 hours, the changes in CRT were observed via immunofluorescence. (B) MKN45 cells were treated with OXA at various concentrations (0,10,20,and 40 μM) for 48 hours.The expression level of CRT on cell membrane was measured using flow cytometry with dead cells,and a statistical chart was generated.(C) MKN45 cells were treated with OXA at various concentrations (0,10,20 and 40 μM) for 48 hours, the content of HMGB1 in the cell supernatant was measured using ELISA, and a statistical chart was generated.(D) AGS cells were treated with OXA at concentrations(0,10,20, and 40 μM) for 48 hours, the ATP content in the supernatant was quantified using ATP assay kit, and a statistical graph was created. The values indicate the mean±standard deviation (SD) of three independent experiments.Scale bar: 100 μm. Statistical significance is indicated as follows: "ns" indicates no statistically significant difference, "*" indicates $P < 0.05$, "**" indicates $P < 0.01$, and "▲" indicates $P < 0.001$.

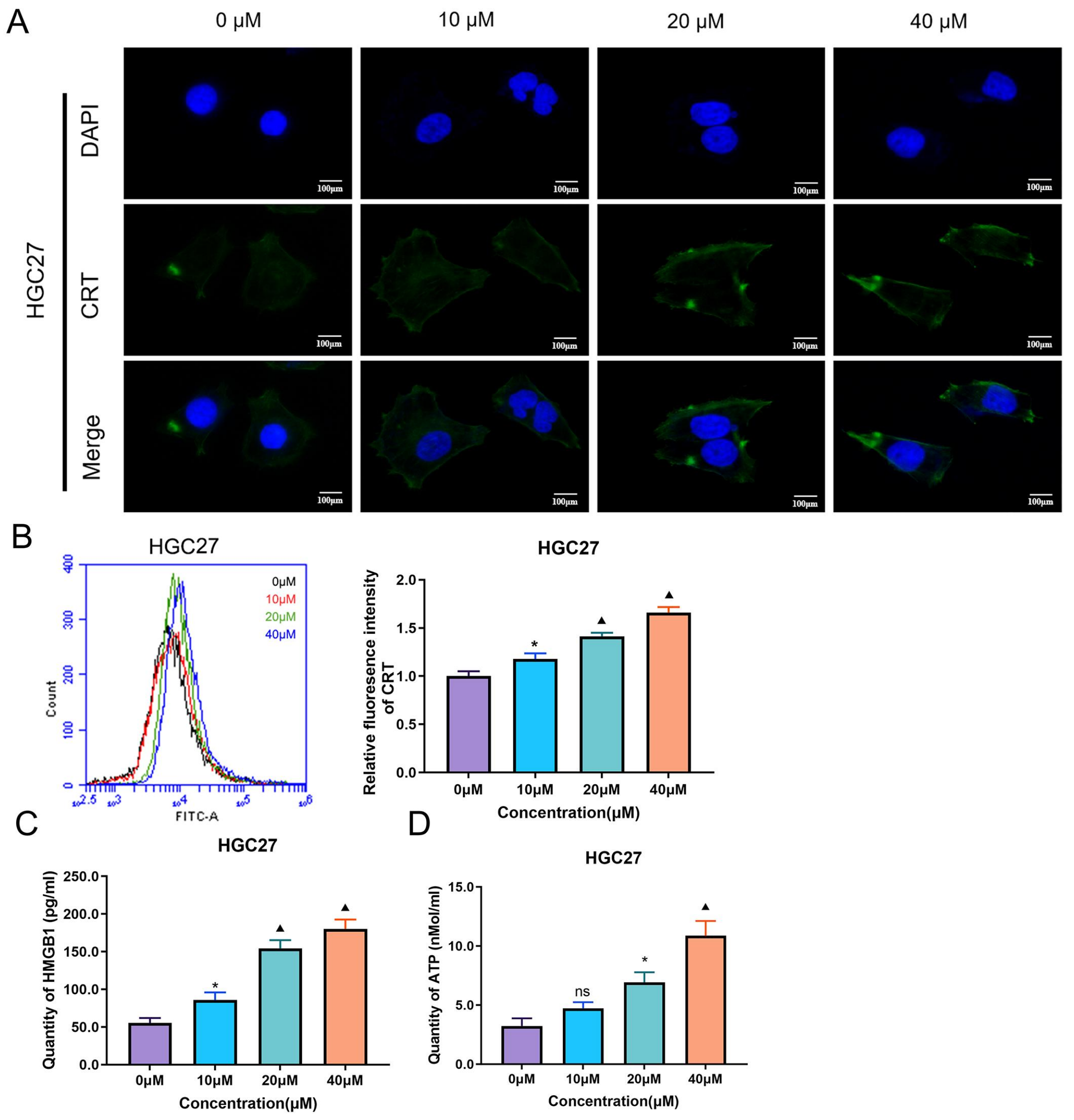

Supplementary Figure 2 OXA was able to induce immunogenic cell death in MKN45 cells

(A) HGC27 cells were treated with OXA at various concentrations (0,10,20,and 40 μM) for 48 hours, the changes in CRT were observed via immunofluorescence. (B) HGC27 cells were treated with OXA at various concentrations (0,10,20,and 40 μM) for 48 hours.The expression level of CRT on cell membrane was measured using flow cytometry with dead cells,and a statistical chart was generated. (C) HGC27 cells were treated with OXA at various concentrations (0,10,20 and 40 μM) for 48 hours, the content of HMGB1 in the cell supernatant was measured using ELISA, and a statistical chart was generated.(D) HGC27 cells were treated with OXA at various concentrations (0,10,20,and 40 μM) for 48 hours, the ATP content in the supernatant was quantified using ATP assay kit, and a statistical graph was created. The values indicate the mean±standard deviation (SD) of three independent experiments.Scale bar: 100 μm. Statistical significance is indicated as follows: "ns" indicates no statistically significant difference, "*" indicates $P < 0.05$, and "▲" indicates $P < 0.001$.

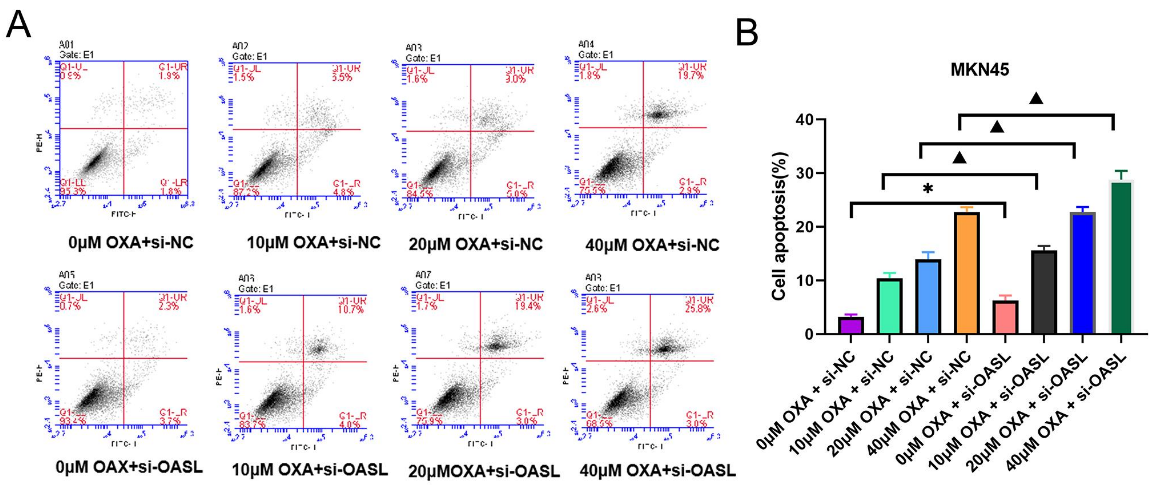

Supplementary Figure 3 Effect of OXA combined with si-OASL on the apoptosis rate in MKN45 cells

(A) MKN45 cells underwent OASL knockdown and were subsequently treated with OXA at various concentrations (0, 10, 20, and 40 μM) for 48 hours, the apoptosis rate was measured by flow cytometry using the Annexin FITC/PI double staining method, and(B) a statistical analysis of the apoptosis rate was conducted. The values indicate the mean±standard deviation (SD) of three independent experiments.Statistical significance is indicated as follows: "*" indicates $P < 0.05$, and "▲" indicates $P < 0.001$.

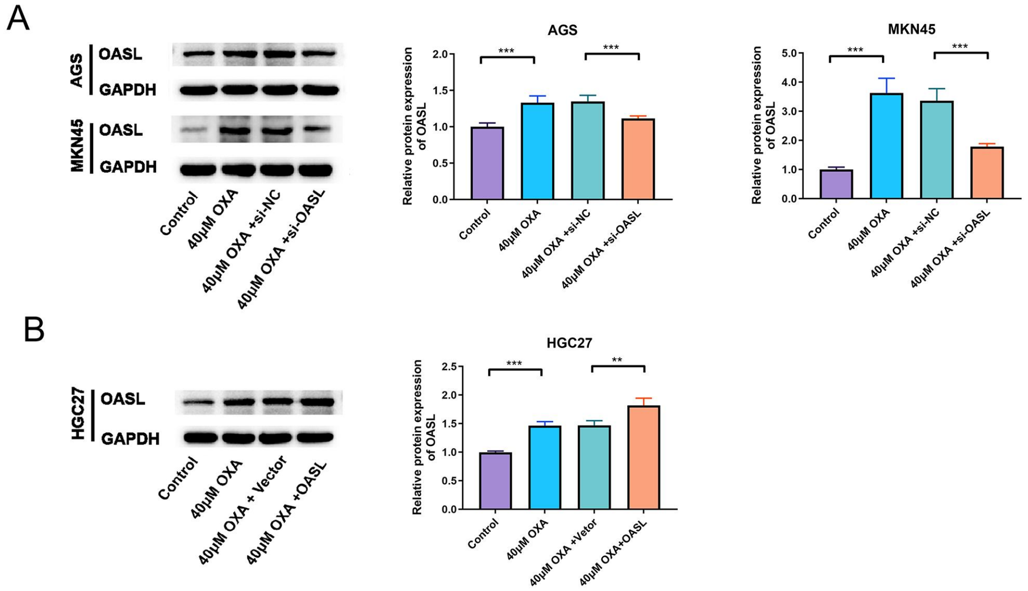

(A) AGS and MKN45 cells were treated with 40 μM OXA combined with si-OASL for 48 hours, and the expression levels of OASL was determined by Western blot,.and statistical graphs were obtained. (B) HGC27 cells were treated with 40 μM OXA in combination with overexpression of OASL for 48 hours, and the expression levels of OASL was determined by Western blot,and statistical graph was obtained. The values indicate the mean±standard deviation (SD) of three independent experiments.Statistical significance is indicated as follows: "**" indicates $P < 0.01$, and "***" indicates $P < 0.001$.

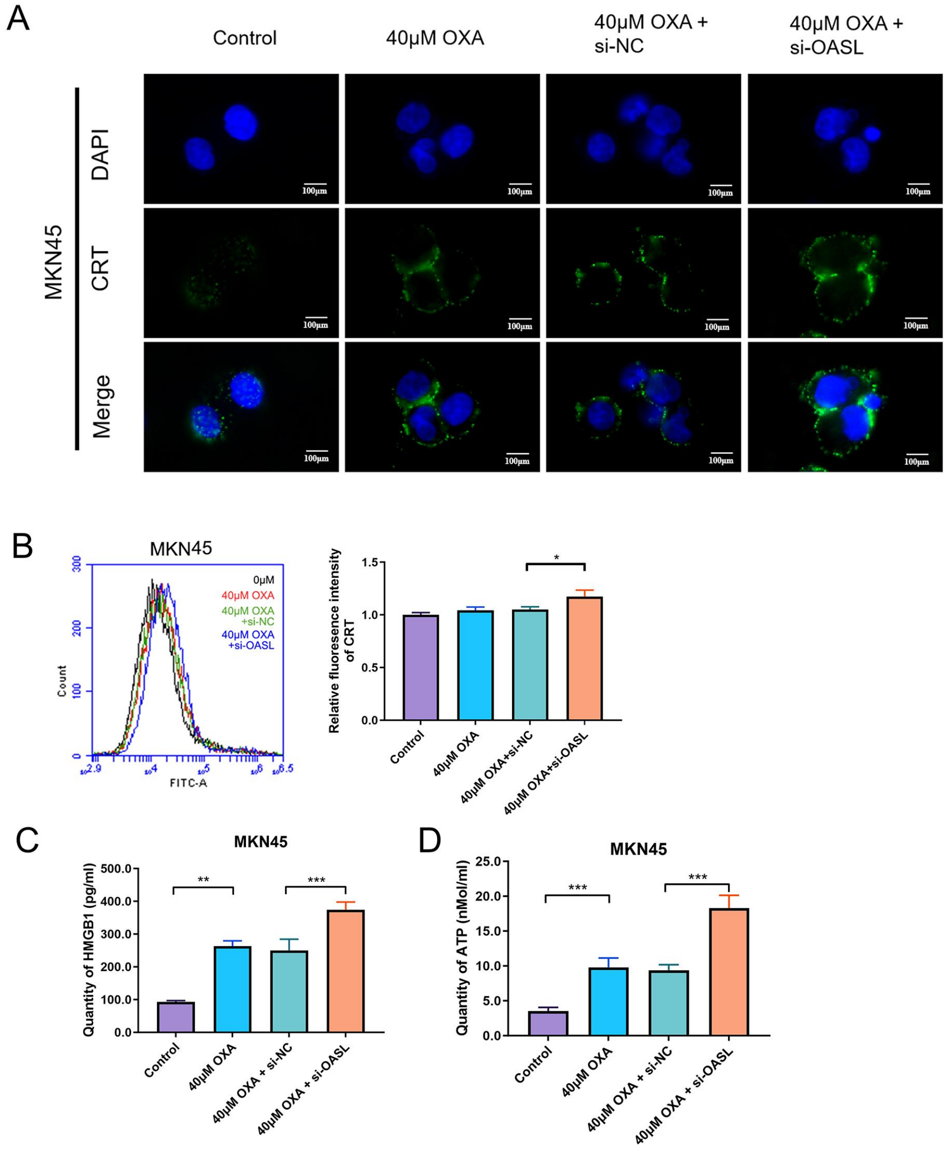

Supplementary Figure 5 OASL was able to reduce OXA-induced immunogenic cell death in MKN45 cells

(A) MKN45 cells were treated with 40 μM OXA combined with si-OASL for 48 hours, and changes in CRT were observed using immunofluorescence. (B) MKN45 cells were treated with 40 μM OXA combined with si-OASL for 48 hours, and the statistical plot of HMGB1 content in cell supernatants was detected using ELISA assay. (C) MKN45 cells were treated with 40 μM OXA combined with si-OASL for 48 hours, and statistical plot of ATP content in cell supernatant detected using ATP kit.The values indicate the mean±standard deviation (SD) of three independent experiments. Scale bar: 100 μm. Statistical significance is indicated as follows: "**" indicates $P < 0.01$, and "***" indicates $P < 0.001$.

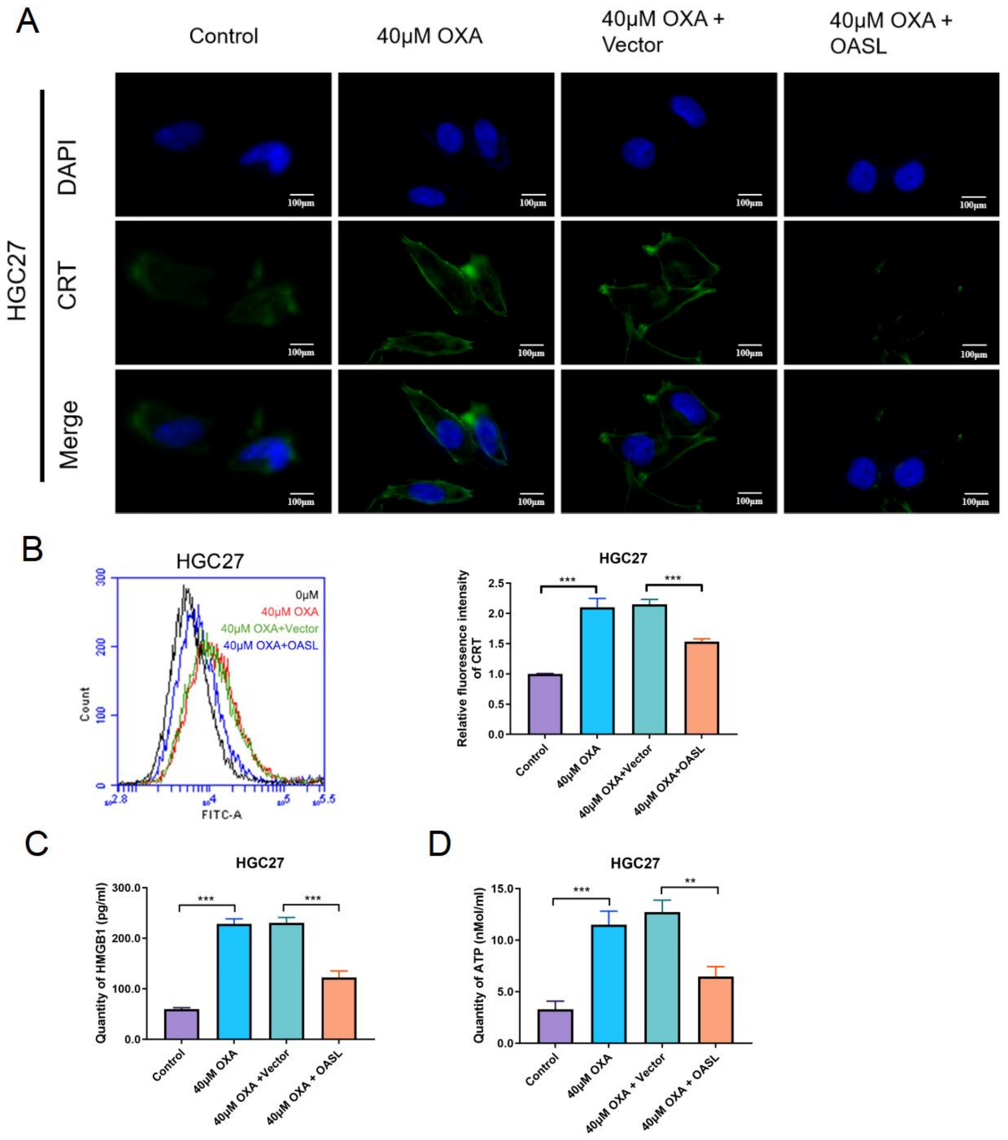

Supplementary Figure S6 OASL was able to reduce OXA-induced immunogenic cell death in HGC27 cells

(A) HGC27 cells were treated with 40 μM OXA combined with overexpressed OASL for 48 hours, and the changes in CRT were observed using immunofluorescence. (B) HGC27 cells were treated with 40 μM OXA combined with overexpressed OASL for 48 hours, and the statistical plot of HMGB1 content in cell supernatants was detected using ELISA assay. (C) HGC27 cells were treated with 40 μM OXA combined with overexpressed OASL for 48 hours, and statistical plot of ATP content in cell supernatant detected using ATP kit. The values indicate the mean±standard deviation (SD) of three independent experiments. Scale bar: 100 μm. Statistical significance is indicated as follows: "**" indicates $P < 0.01$, and "***" indicates $P < 0.001$.

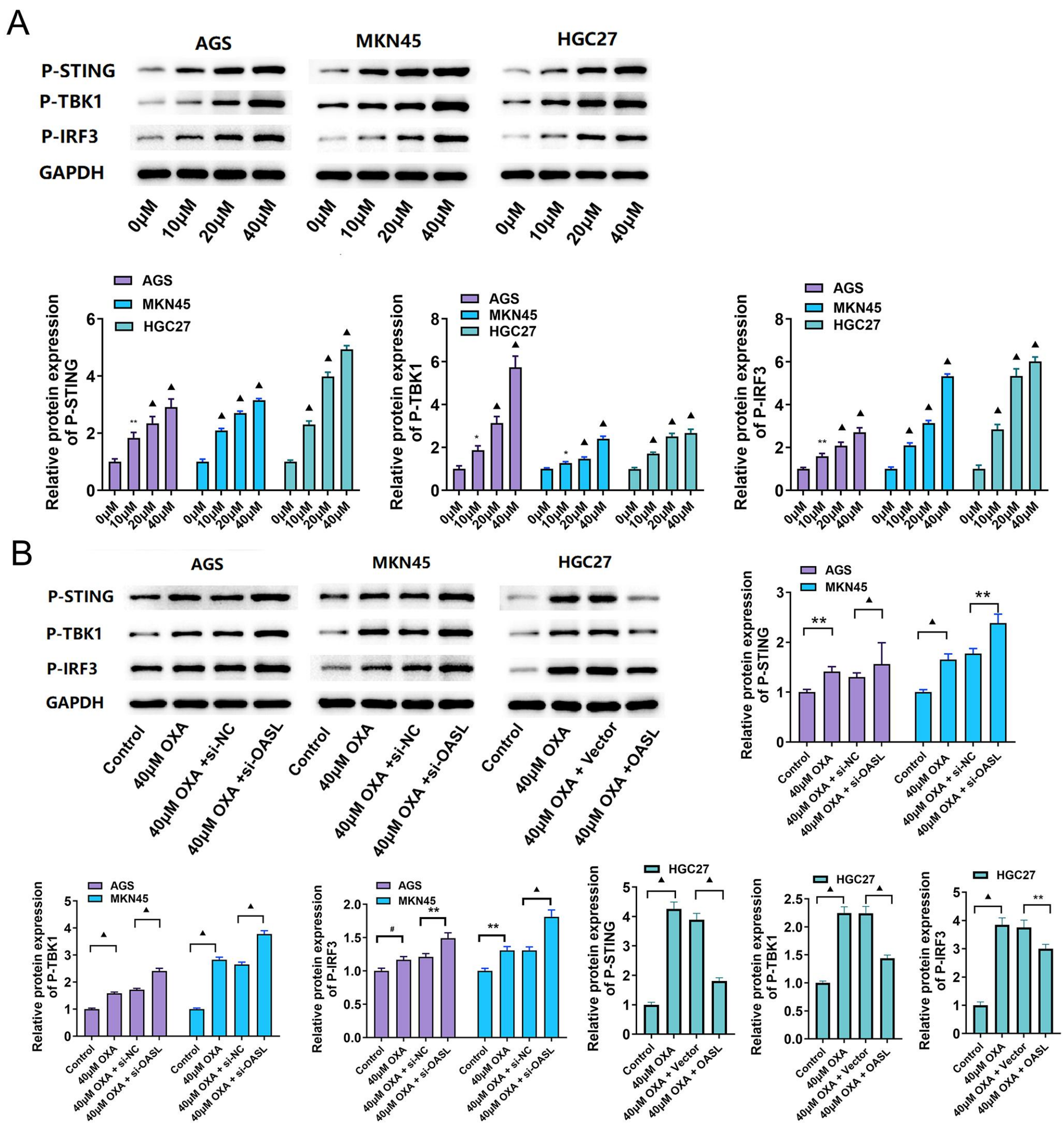

Supplementary Figure 7 OASL regulates the expression of key proteins of the cGAS-STING signaling pathway

(A) AGS,MKN45,and HGC27 cells were treated with OXA at various concentrations (0,10,20, and 40 μM) for 48 hours, and the expression levels of key proteins of the cGAS-STING signaling pathway were determined by western blot, and statistical graphs were obtained.(B) AGS and MKN45 cells were treated with 40 μM OXA combined with si-OASL for 48 hours, and the expression levels of key proteins of the cGAS-STING signaling pathway were determined by western blot,and statistical graphs were obtained. HGC27 cells were treated with 40 μM OXA in combination with overexpression of OASL for 48 hours, and the expression levels of key proteins of the cGAS-STING signaling pathway were determined by western blot,and statistical graphs were obtained. The values indicate the mean±standard deviation (SD) of three independent experiments. Statistical significance is indicated as follows: "*" indicates P < 0.05,"**" indicates $P < 0.01$, and "▲" indicates $P < 0.001$.

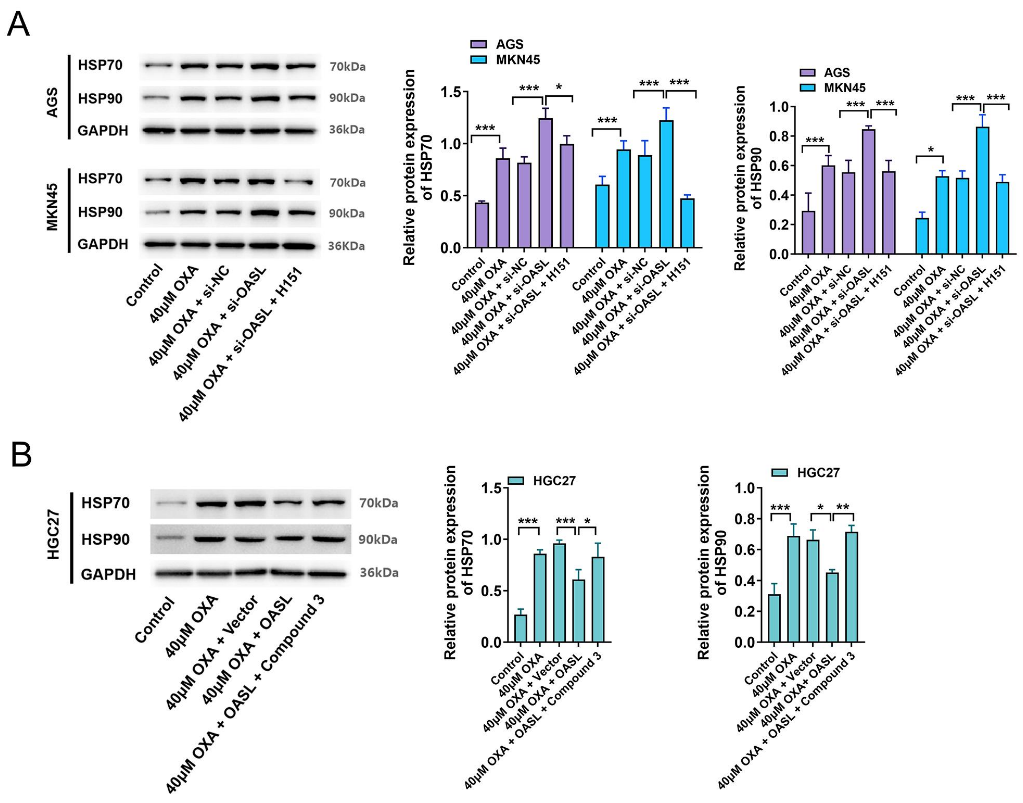

Supplementary Figure 8 OASL relies on cGAS-STING signaling pathway to regulate OXA-induced immunogenic cell death

(A) AGS and MKN45 cells were treated with 40 μM OXA in combination with si-OASL and supplemented with H151(an inhibitor of the cGAS-STING signaling pathway) for 48 hours, and the expression levels of HSP70 and HSP90 were determined by Western blot,.and statistical graphs were obtained.(B) HGC27 cells were treated with 40 μM OXA combined with overexpressed OASL and supplemented with Compound 3 (an activator of the cGAS-STING signaling pathway) for 48 hours, the expression levels of HSP70 and HSP90 were determined by Western blot,.and statistical graphs were obtained.The values indicate the mean±standard deviation (SD) of three independent experiments. Statistical significance is indicated as follows: "*" indicates $P < 0.05$,"**" indicates $P < 0.01$, and"***" indicates $P < 0.001$.

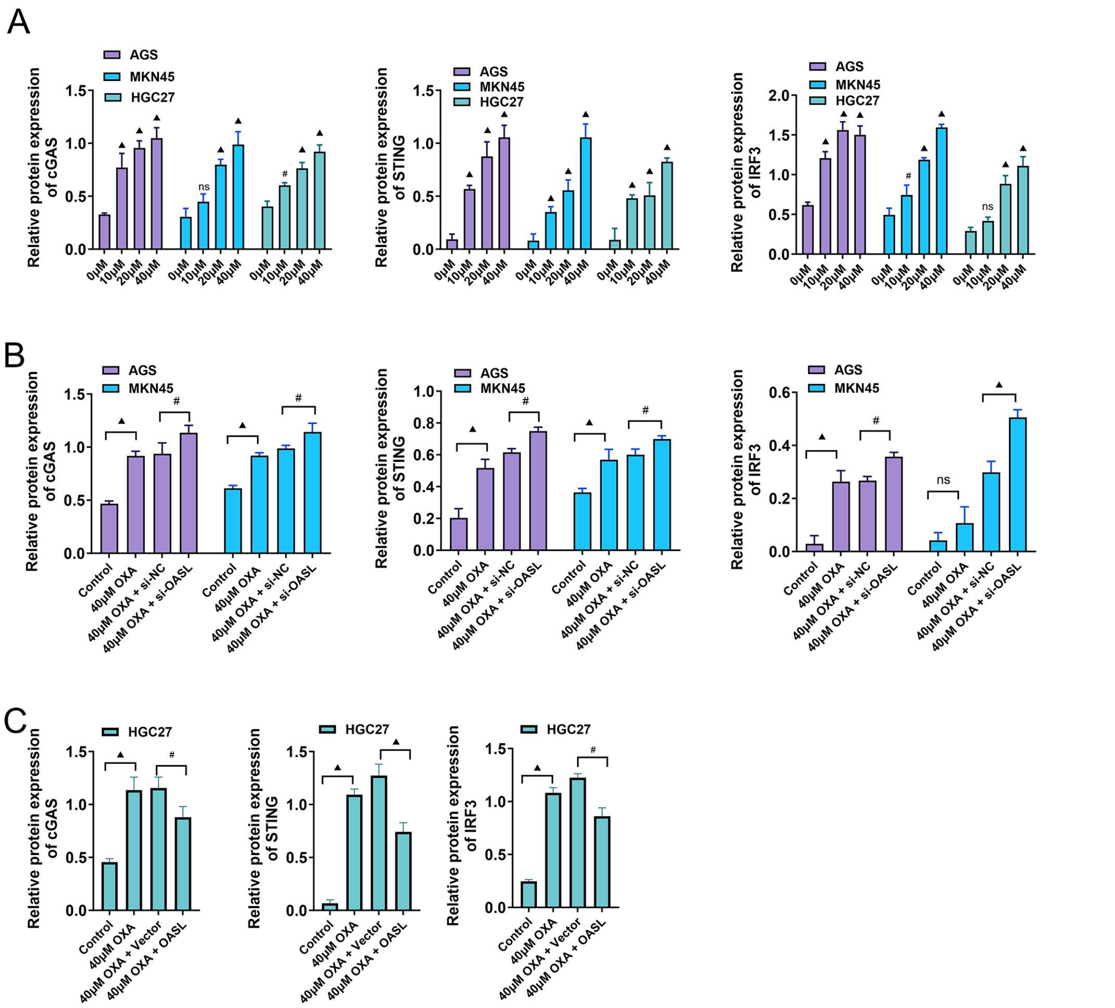

Supplementary Figure 9 OASL regulates the expression of key proteins in the cGAS-STING signaling pathway

(A) AGS, MKN45, and HGC27 cells were treated with OXA at various concentrations (0,10,20, and 40 μM) for 48 hours, and the expression levels of key proteins of the cGAS-STING signaling pathway were determined by Western blot, and statistical graphs were obtained.(B) AGS and MKN45 cells were treated with 40 μM OXA combined with si-OASL for 48 hours, and the expression levels of key proteins of the cGAS-STING signaling pathway were determined by Western blot,.and statistical graphs were obtained. (C) HGC27 cells were treated with 40 μM OXA in combination with overexpression of OASL for 48 hours, and the expression levels of key proteins of the cGAS-STING signaling pathway were determined by Western blot,and statistical graphs were obtained. The values indicate the mean±standard deviation (SD) of three independent experiments.Statistical significance is indicated as follows: "ns" indicates no statistically significant difference, "#" indicates $P < 0.05$, and "▲" indicates $P < 0.001$.

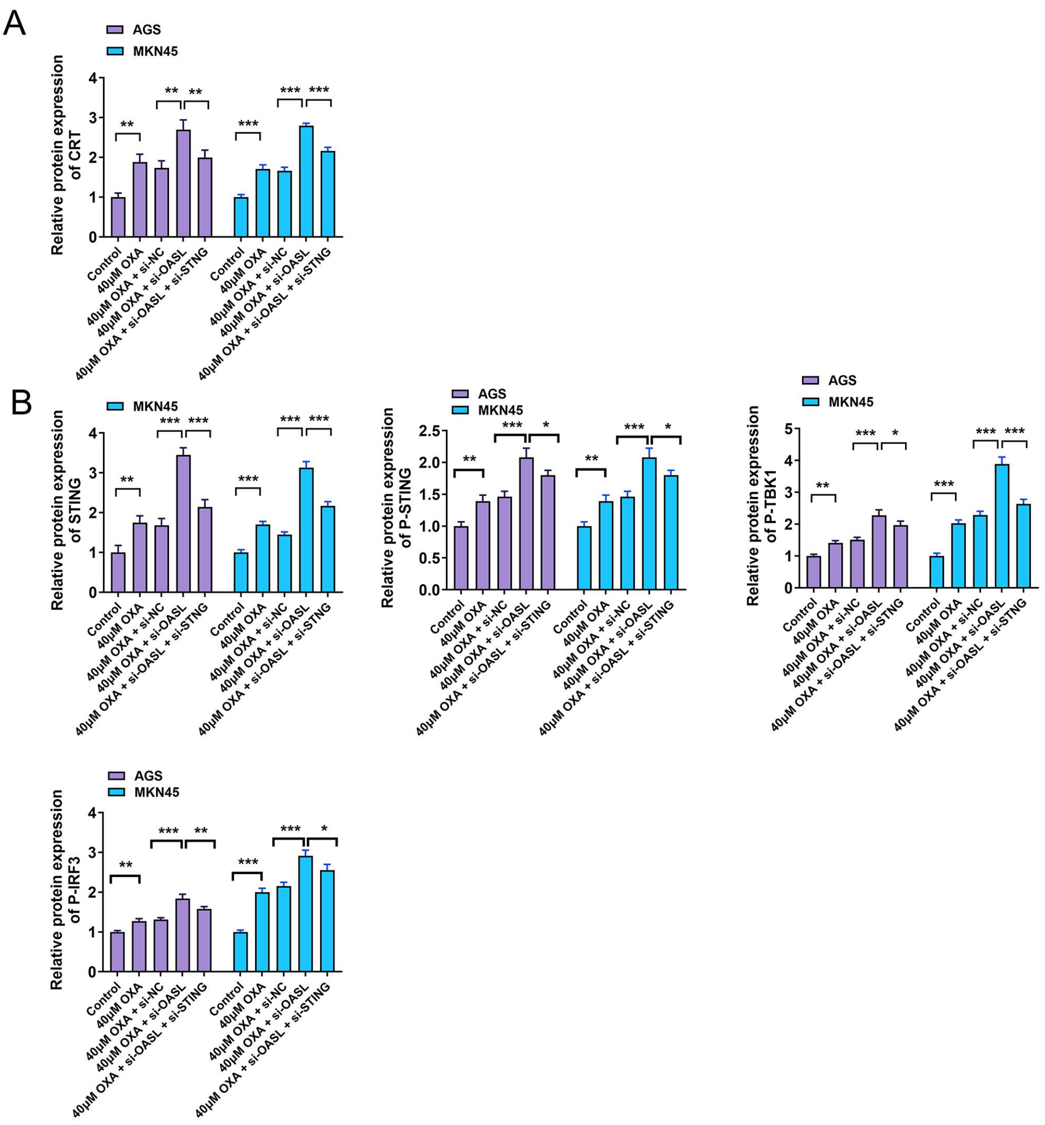

Supplementary Figure 10 OASL-dependent cGAS-STING signaling pathway to regulate OXA-induced immunogenic cell death

(A) AGS and MKN 45 cells were treated with 40 μM OXA in combination with si-OASL and si-STING for 48 hours,the expression levels of CRT protein in AGS and MKN45 cells were determined by Western blot,and statistical graphs were obtained. (B) AGS and MKN 45 cells were treated with 40 μM OXA in combination with si-OASL and si-STING for 48 hours, and the expression levels of key proteins of the cGAS-STING signaling pathway were determined by Western blot,and statistical graphs were obtained.The values indicate the mean±standard deviation (SD) of three independent experiments.Statistical significance is indicated as follows: "*" indicates $P < 0.05$, "**" indicates $P < 0.01$, and "***" indicates $P < 0.001$.

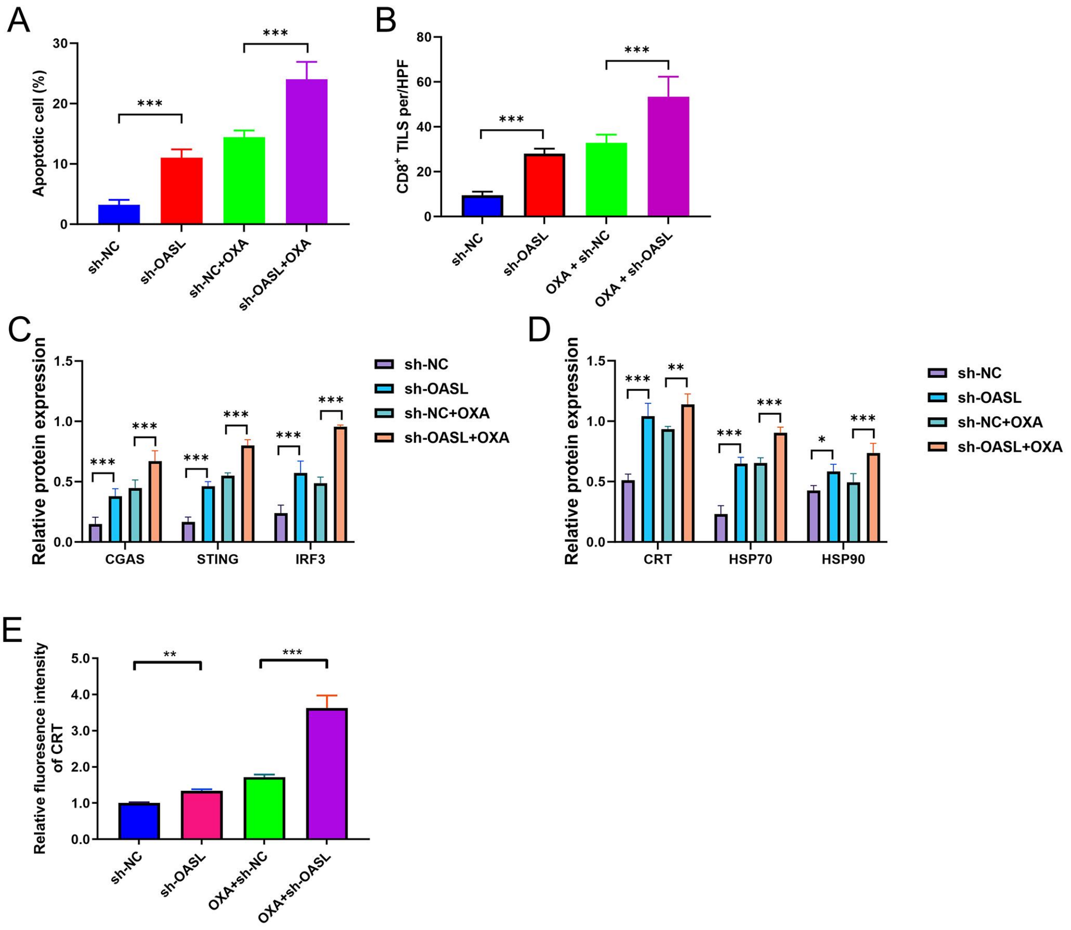

Supplementary Figure 11 A mouse tumor-bearing model verified that knockdown of OASL improved OXA-induced ICD in GC cells

(A) Apoptosis and statistics of mouse tumor-bearing tumors detected by TUNEL staining, and statistical graphs were obtained. (B) CD8 expression and statistics of mouse tumor bearing by immunohistochemical fluorescence staining,and statistical graphs were obtained.(C) Expression and statistical plots of key proteins of cGAS-STING signaling pathway determined by Western blot,.and statistical graphs were obtained. (D) Expression and statistical plots of the CRT and HSP70 and HSP90 proteins associated with ICD were determined by western blot,and statistical graphs were obtained.(E) The expression level of CRT on cell membrane was measured using flow cytometry with dead cells,and a statistical chart was generated. The values indicate the mean±standard deviation (SD) of three independent experiments.Statistical significance is indicated as follows: "*" indicates $P < 0.05$, "**" indicates $P < 0.01$, and "***" indicates $P < 0.001$
