# Supplementary material for: Effect of OASL on oxaliplatin-induced immunogenic cell death in gastric cancer via the cGAS-STING signaling pathway
Source: Cell Death Discov. 2025 Nov 21;12:20. doi: 10.1038/s41420-025-02850-w (PMC12804755; doi:10.1038/s41420-025-02850-w)
Supplement: Supplementary file 2 — WB [file 41420_2025_2850_MOESM2_ESM.pdf]

Fig 2E

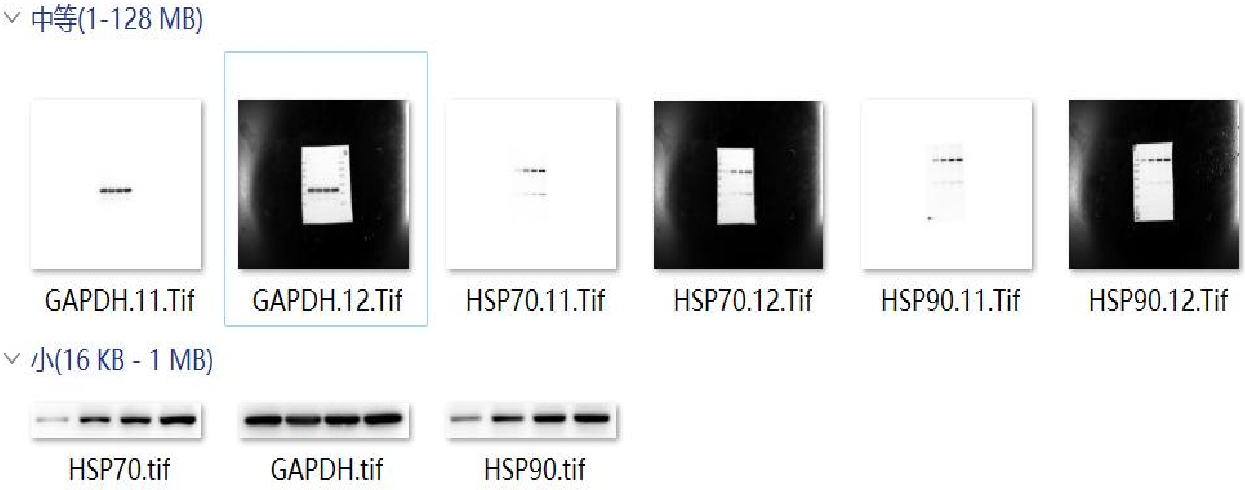
中等(1-128 MB)
GAPDH.11.Tif
GAPDH.12.Tif
HSP70.11.Tif
HSP70.12.Tif
HSP90.11.Tif
HSP90.12.Tif
小(16 KB - 1 MB)
HSP70.tif
GAPDH.tif
HSP90.tif

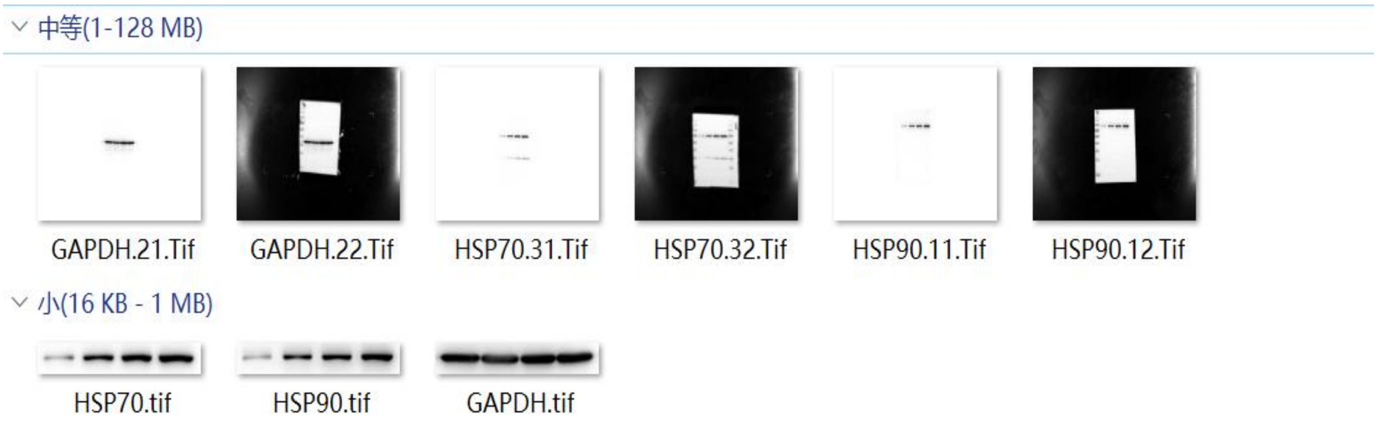
中等(1-128 MB)
GAPDH.21.Tif
GAPDH.22.Tif
HSP70.31.Tif
HSP70.32.Tif
HSP90.11.Tif
HSP90.12.Tif
小(16 KB - 1 MB)
HSP70.tif
HSP90.tif
GAPDH.tif

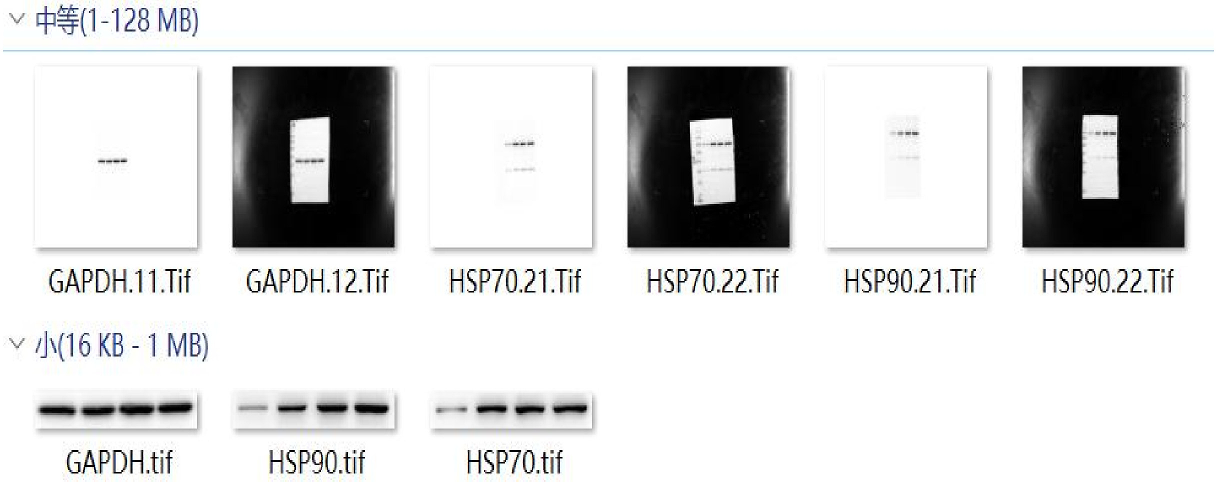
中等(1-128 MB)
GAPDH.11.Tif
GAPDH.12.Tif
HSP70.21.Tif
HSP70.22.Tif
HSP90.21.Tif
HSP90.22.Tif
小(16 KB - 1 MB)
GAPDH.tif
HSP90.tif
HSP70.tif

Fig3A

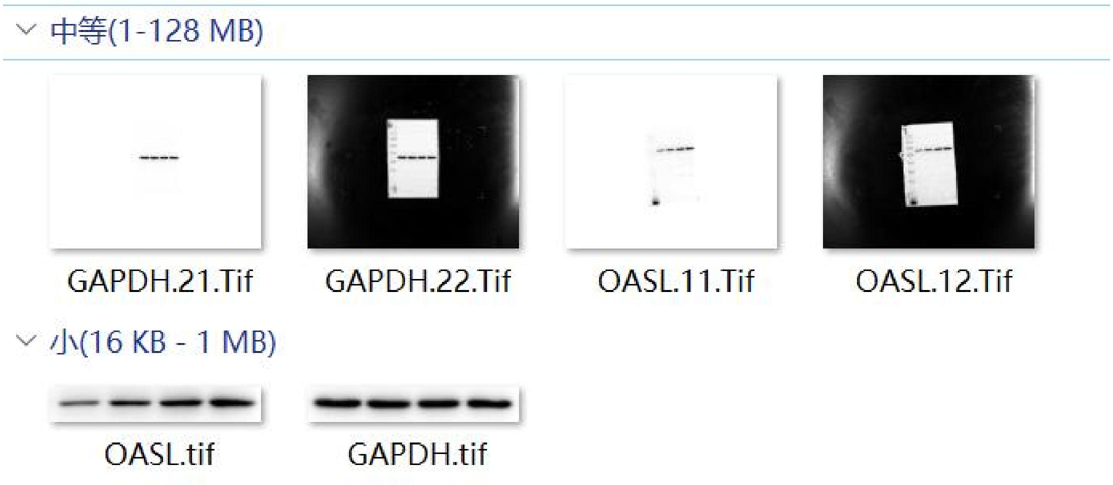
中等(1-128 MB)
GAPDH.21.Tif
GAPDH.22.Tif
OASL.11.Tif
OASL.12.Tif
小(16 KB - 1 MB)
OASL.tif
GAPDH.tif

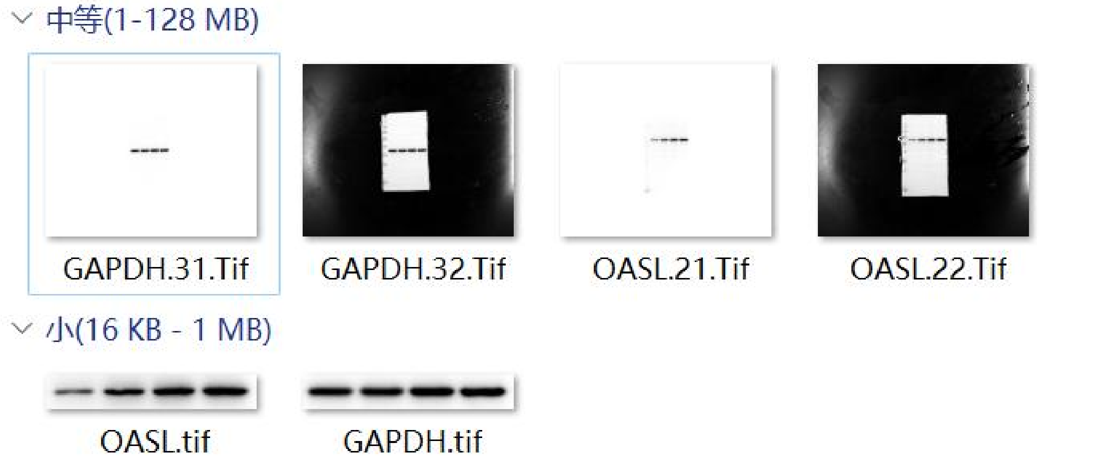
中等(1-128 MB)
GAPDH.31.Tif
GAPDH.32.Tif
OASL.21.Tif
OASL.22.Tif
小(16 KB - 1 MB)
OASL.tif
GAPDH.tif

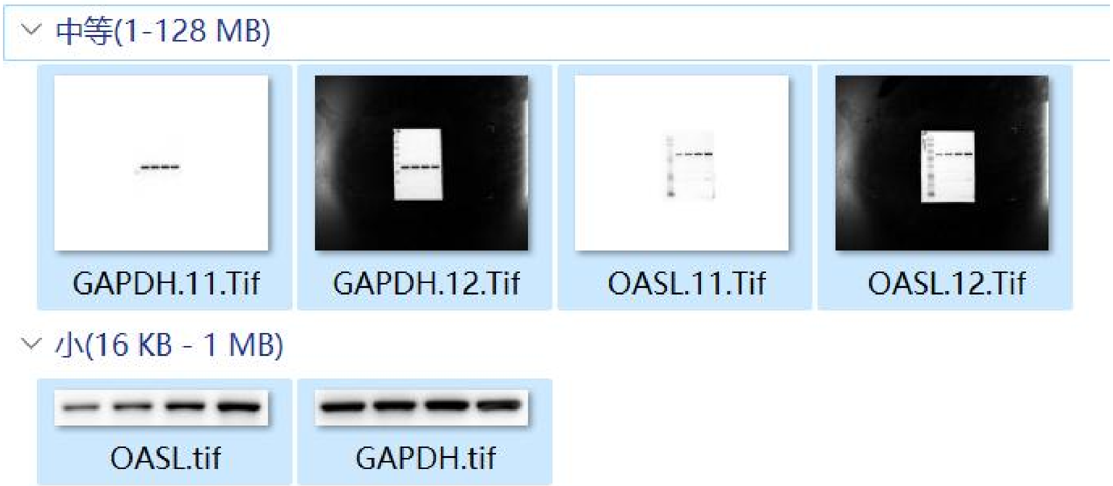
中等(1-128 MB)
GAPDH.11.Tif
GAPDH.12.Tif
OASL.11.Tif
OASL.12.Tif
小(16 KB - 1 MB)
OASL.tif
GAPDH.tif

Fig-4E

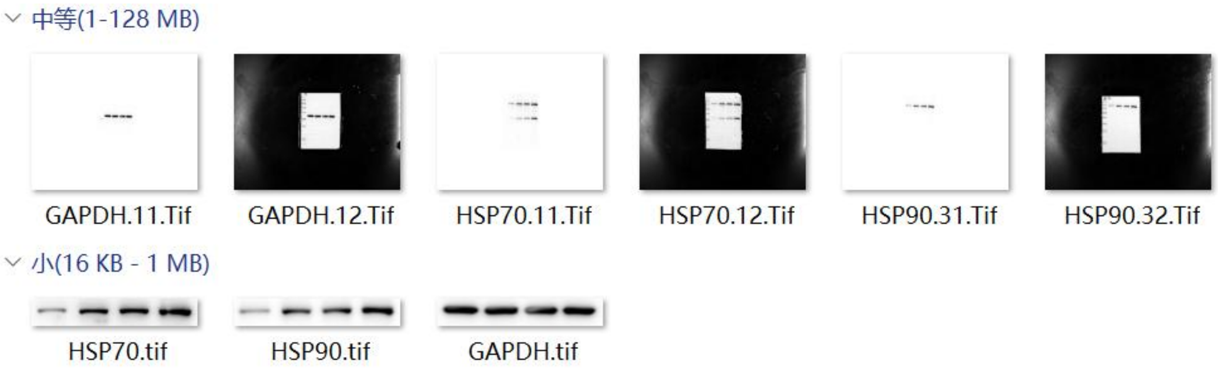
中等(1-128 MB)
GAPDH.11.Tif
GAPDH.12.Tif
HSP70.11.Tif
HSP70.12.Tif
HSP90.31.Tif
HSP90.32.Tif
小(16 KB - 1 MB)
HSP70.tif
HSP90.tif
GAPDH.tif

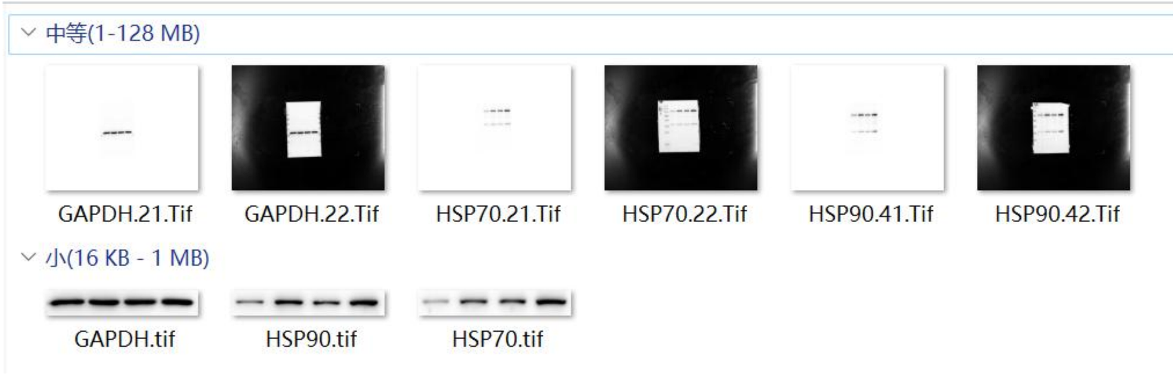
中等(1-128 MB)
GAPDH.21.Tif
GAPDH.22.Tif
HSP70.21.Tif
HSP70.22.Tif
HSP90.41.Tif
HSP90.42.Tif
小(16 KB - 1 MB)
GAPDH.tif
HSP90.tif
HSP70.tif

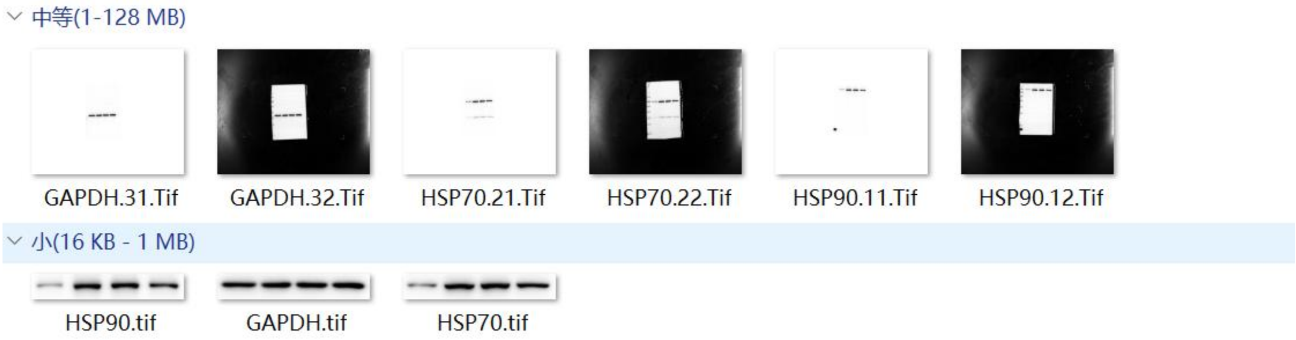
中等(1-128 MB)
GAPDH.31.Tif
GAPDH.32.Tif
HSP70.21.Tif
HSP70.22.Tif
HSP90.11.Tif
HSP90.12.Tif
小(16 KB - 1 MB)
HSP90.tif
GAPDH.tif
HSP70.tif

Fig 5D-F

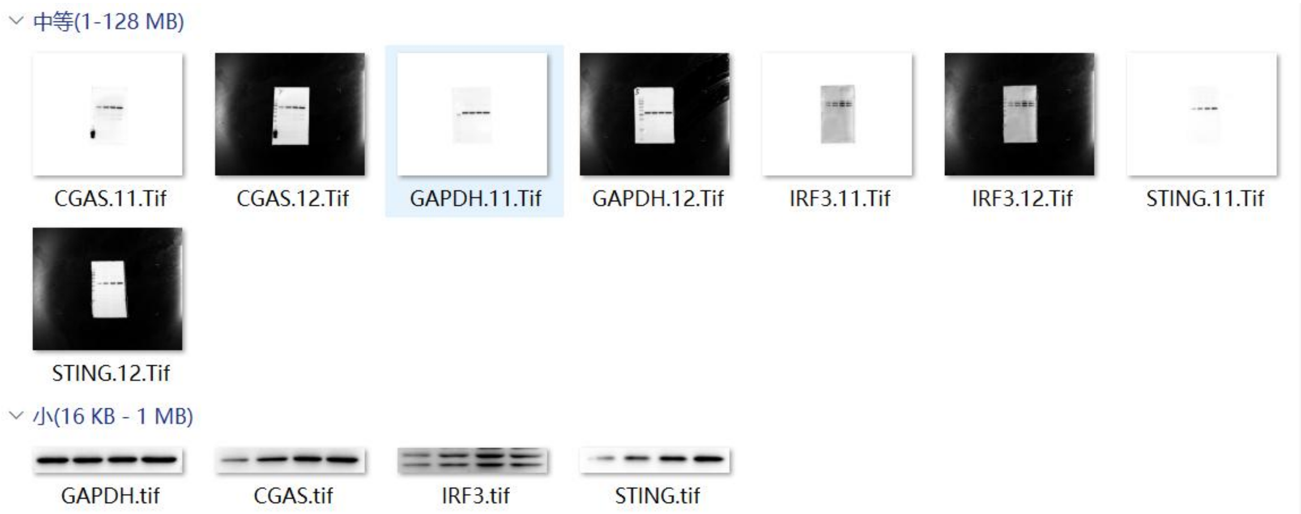
中等(1-128 MB)
CGAS.11.Tif
CGAS.12.Tif
GAPDH.11.Tif
GAPDH.12.Tif
IRF3.11.Tif
IRF3.12.Tif
STING.11.Tif
STING.12.Tif
小(16 KB - 1 MB)
GAPDH.tif
CGAS.tif
IRF3.tif
STING.tif

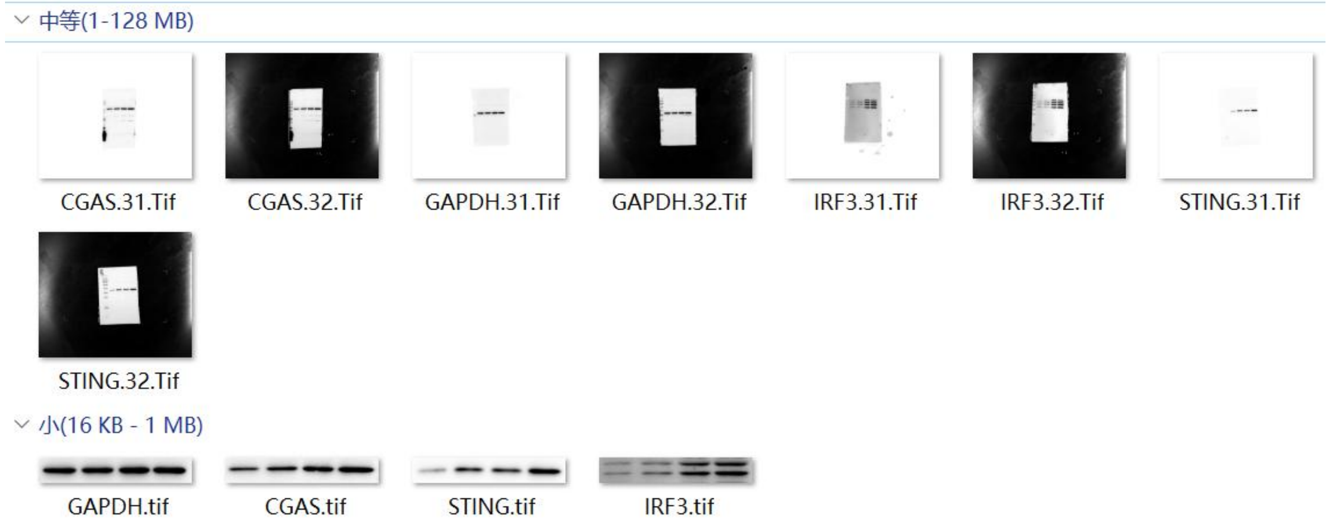
中等(1-128 MB)
CGAS.31.Tif
CGAS.32.Tif
GAPDH.31.Tif
GAPDH.32.Tif
IRF3.31.Tif
IRF3.32.Tif
STING.31.Tif
STING.32.Tif
小(16 KB - 1 MB)
GAPDH.tif
CGAS.tif
STING.tif
IRF3.tif

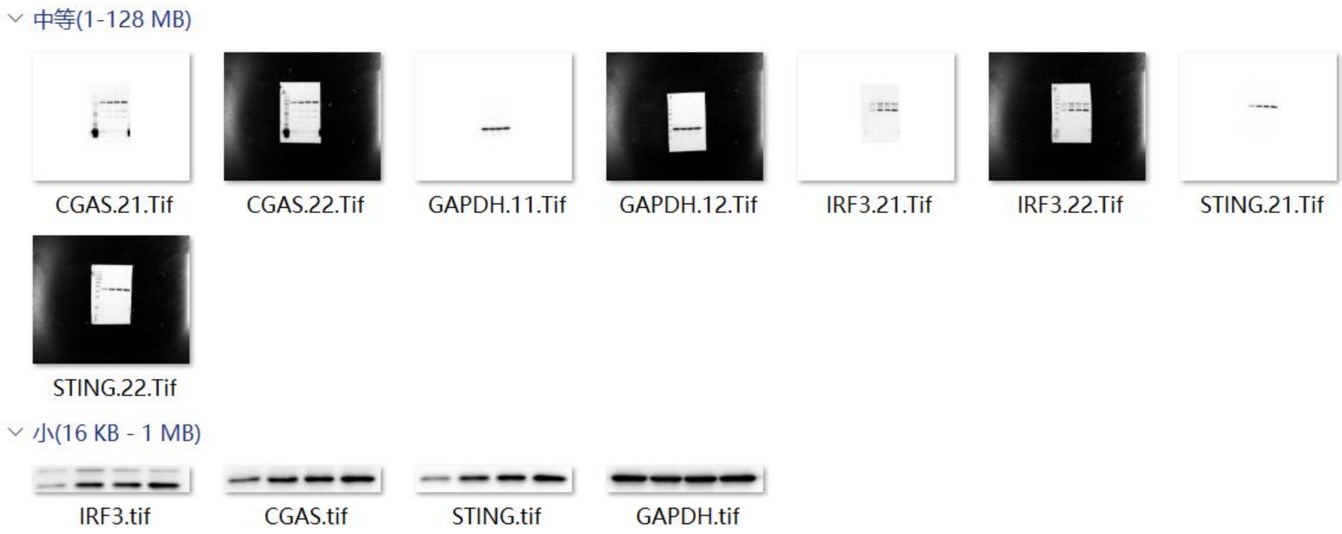
中等(1-128 MB)
CGAS.21.Tif
CGAS.22.Tif
GAPDH.11.Tif
GAPDH.12.Tif
IRF3.21.Tif
IRF3.22.Tif
STING.21.Tif
STING.22.Tif
小(16 KB - 1 MB)
IRF3.tif
CGAS.tif
STING.tif
GAPDH.tif

中等(1-128 MB)

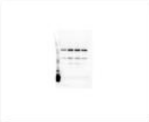
CGAS.11.Tif

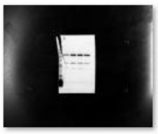
CGAS.12.Tif


GAPDH.31.Tif

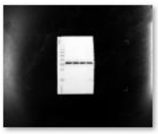
GAPDH.32.Tif

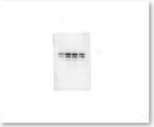
IRF3.11.Tif

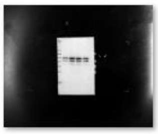
IRF3.12.Tif


STING.11.Tif

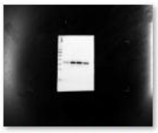
STING.12.Tif

Fig 6A

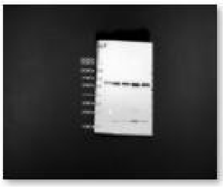
CRT..Tif

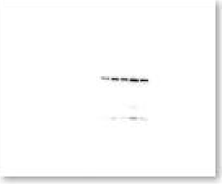
CRT.Tif

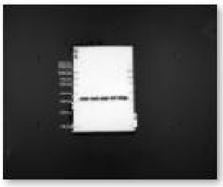
GAPDH..Tif


GAPDH.Tif

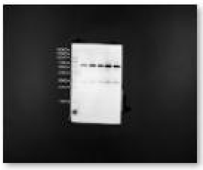
CRT..Tif

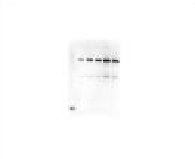
CRT.Tif

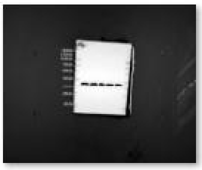
GAPDH..Tif


GAPDH.Tif

Fig 6D

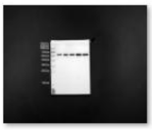
CRT..Tif

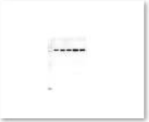
CRT.Tif

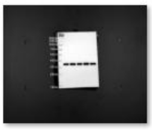
GAPDH..Tif


GAPDH.Tif

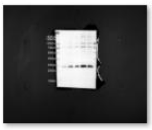
HMGB1..Tif

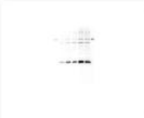
HMGB1.Tif

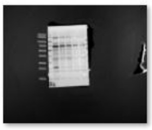
OASL..Tif

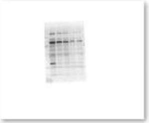
OASL.Tif

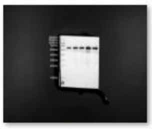
P-IRF3..Tif


P-IRF3.Tif

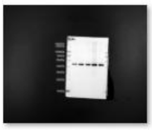
P-STING..Tif

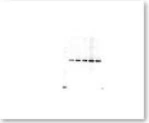
P-STING.Tif

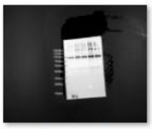
P-TBK1..Tif

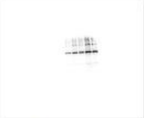
P-TBK1.Tif

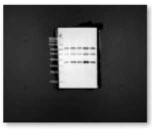
STING..Tif

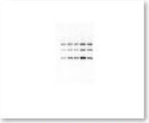
STING.Tif

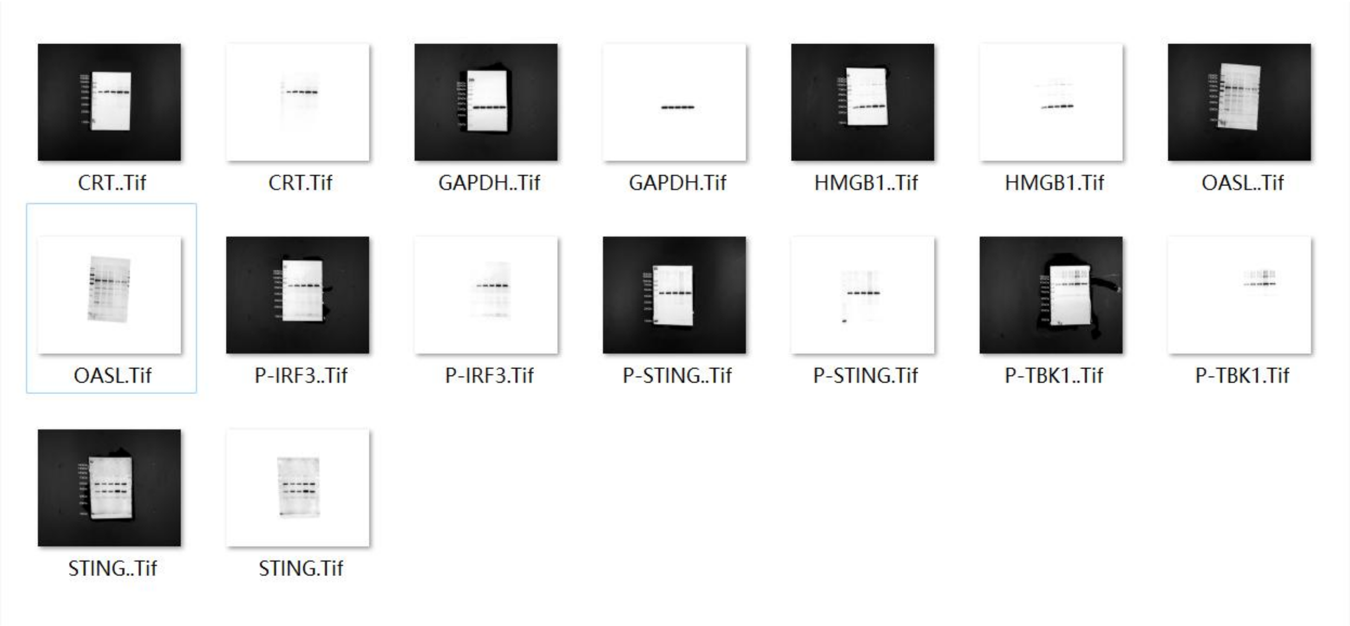
CRT..Tif
CRT.Tif
GAPDH..Tif
GAPDH.Tif
HMGB1..Tif
HMGB1.Tif
OASL..Tif
OASL.Tif
P-IRF3..Tif
P-IRF3.Tif
P-STING..Tif
P-STING.Tif
P-TBK1..Tif
P-TBK1.Tif
STING..Tif
STING.Tif

Fig-7A

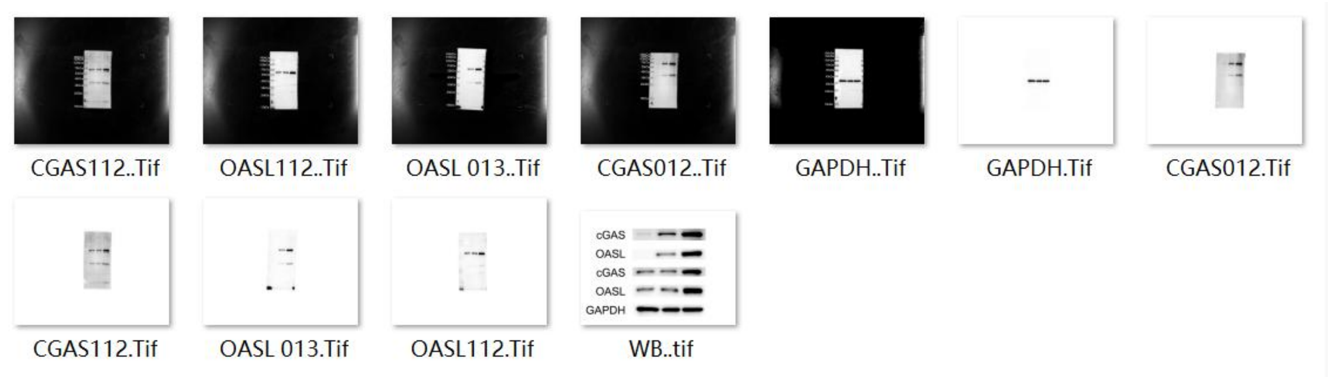
CGAS112..Tif
OASL112..Tif
OASL 013..Tif
CGAS012..Tif
GAPDH..Tif
GAPDH.Tif
CGAS012.Tif
CGAS112.Tif
OASL 013.Tif
OASL112.Tif
cGAS
OASL
cGAS
OASL
GAPDH
WB..tif

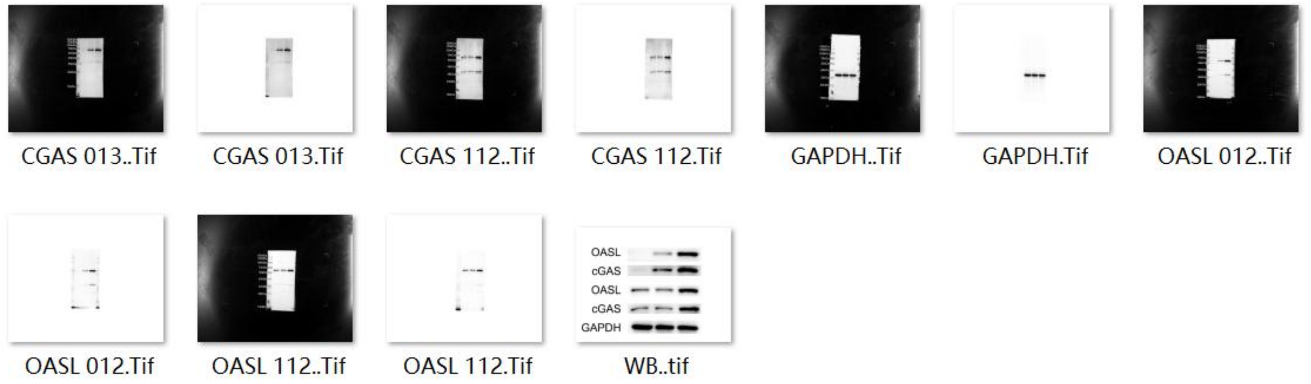
CGAS 013..Tif
CGAS 013.Tif
CGAS 112..Tif
CGAS 112.Tif
GAPDH..Tif
GAPDH.Tif
OASL 012..Tif
OASL 012.Tif
OASL 112..Tif
OASL 112.Tif
OASL
cGAS
OASL
cGAS
GAPDH
WB..tif

Fig-8F-G

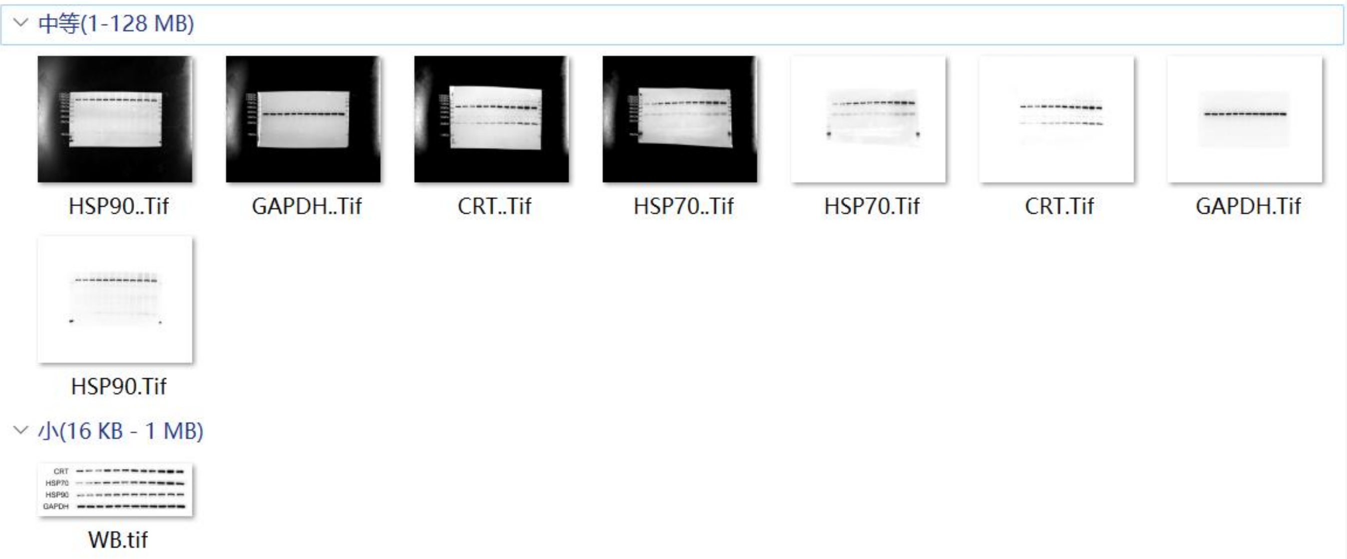
中等(1-128 MB)
HSP90..Tif
GAPDH..Tif
CRT..Tif
HSP70..Tif
HSP70.Tif
CRT.Tif
GAPDH.Tif
HSP90.Tif
小(16 KB - 1 MB)
CRT
HSP70
HSP90
GAPDH
WB.tif

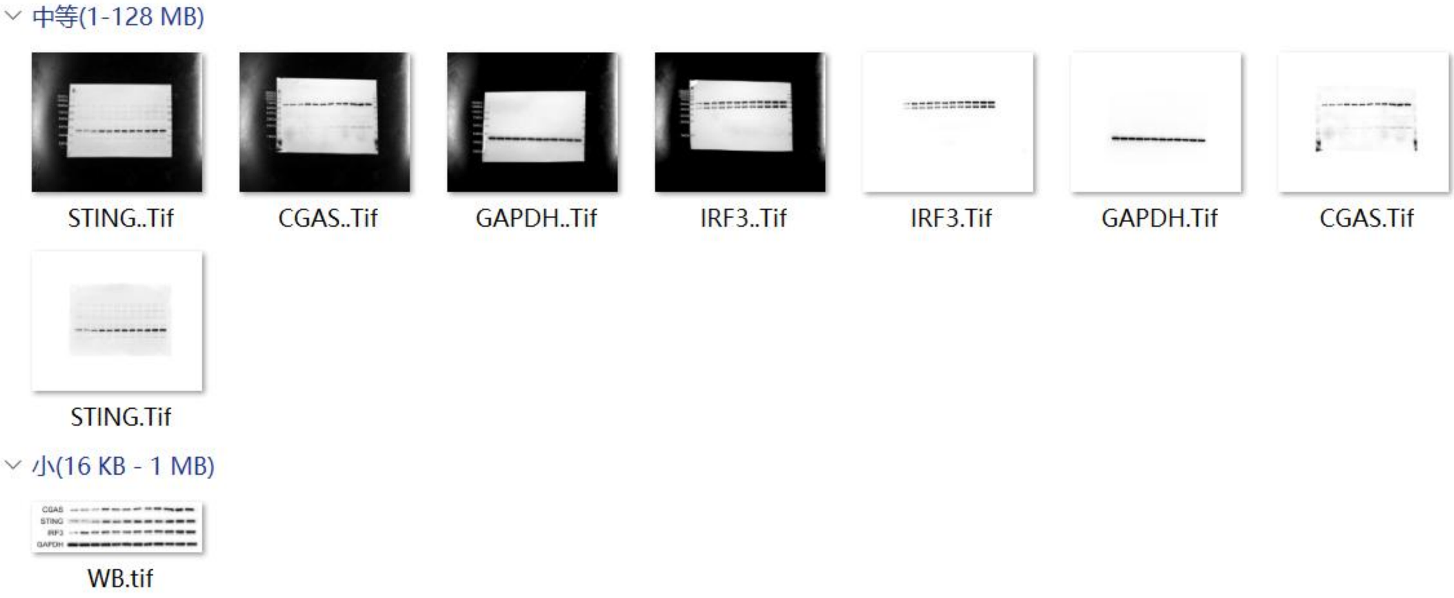
中等(1-128 MB)
STING..Tif
CGAS..Tif
GAPDH..Tif
IRF3..Tif
IRF3.Tif
GAPDH.Tif
CGAS.Tif
STING.Tif
小(16 KB - 1 MB)
CGAS
STING
IRF3
GAPDH
WB.tif

SFig4 A-B

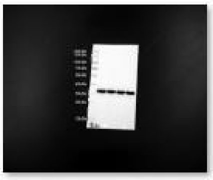
GAPDH..Tif

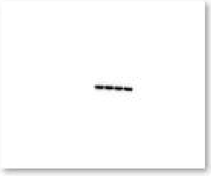
GAPDH.Tif

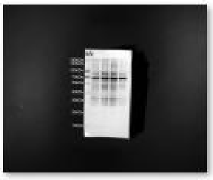
OASL..Tif

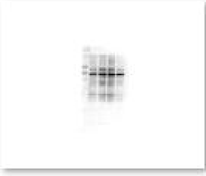
OASL.Tif

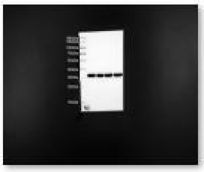
GAPDH..Tif


GAPDH.Tif

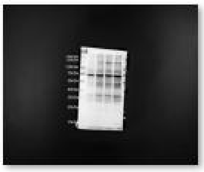
OASL..Tif

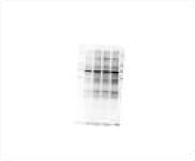
OASL.Tif

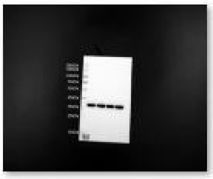
GAPDH..Tif

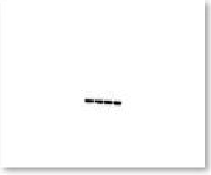
GAPDH.Tif

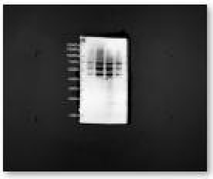
OASL..Tif

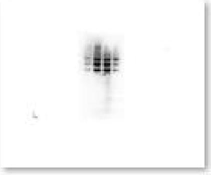
OASL.Tif

SFig7 A

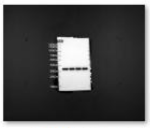
GAPDH..Tif


GAPDH.Tif

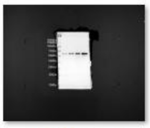
P-IRF3..Tif

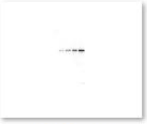
P-IRF3.Tif

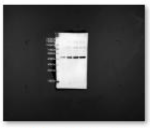
P-STING..Tif

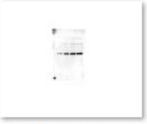
P-STING.Tif

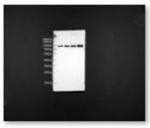
P-TBK1..Tif


P-TBK1.Tif

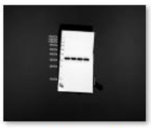
GAPDH..Tif
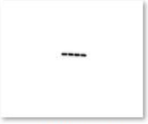
GAPDH.Tif
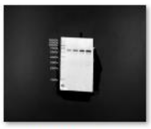
P-IRF3..Tif

P-IRF3.Tif
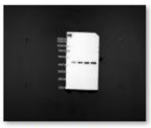
P-STING..Tif
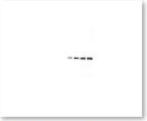
P-STING.Tif
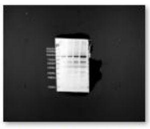
P-TBK1..Tif
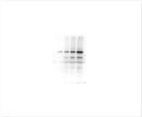
P-TBK1.Tif

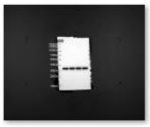
GAPDH..Tif

GAPDH.Tif
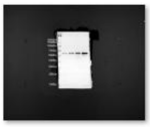
P-IRF3..Tif
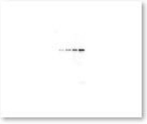
P-IRF3.Tif
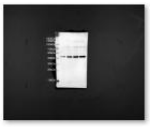
P-STING..Tif
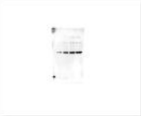
P-STING.Tif
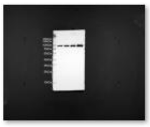
P-TBK1..Tif
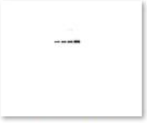
P-TBK1.Tif

SFig7 B

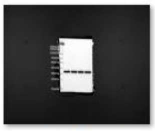
GAPDH..Tif
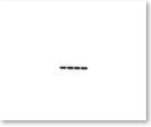
GAPDH.Tif
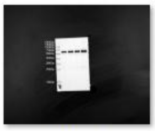
P-IRF3..Tif
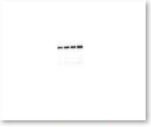
P-IRF3.Tif
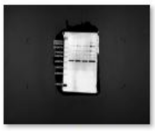
P-STING..Tif
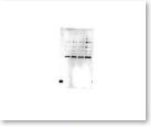
P-STING.Tif
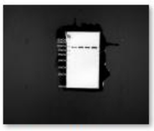
P-TBK1..Tif

P-TBK1.Tif

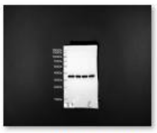
GAPDH..Tif

GAPDH.Tif
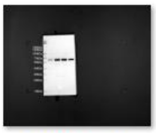
P-IRF3..Tif

P-IRF3.Tif
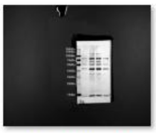
P-STING..Tif
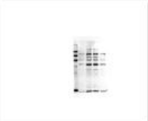
P-STING.Tif
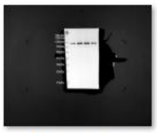
P-TBK1..Tif
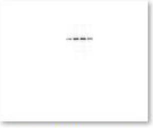
P-TBK1.Tif

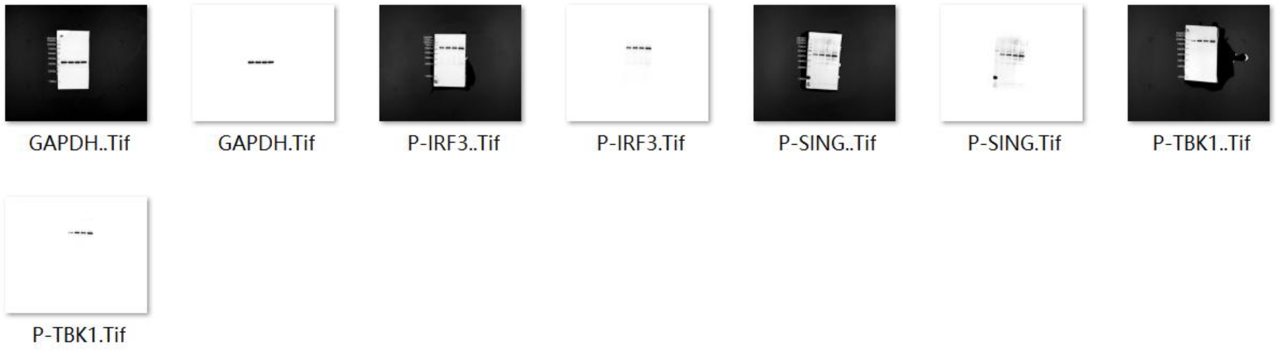
GAPDH..Tif
GAPDH.Tif
P-IRF3..Tif
P-IRF3.Tif
P-SING..Tif
P-SING.Tif
P-TBK1..Tif
P-TBK1.Tif

SFig 8A-B

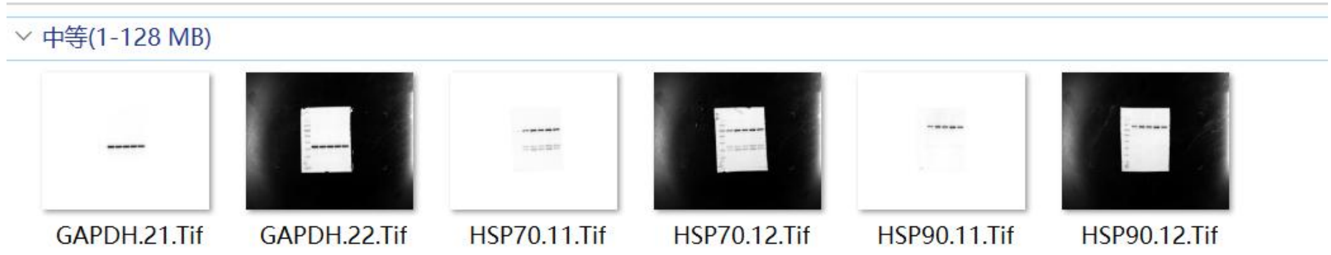
中等(1-128 MB)
GAPDH.21.Tif
GAPDH.22.Tif
HSP70.11.Tif
HSP70.12.Tif
HSP90.11.Tif
HSP90.12.Tif

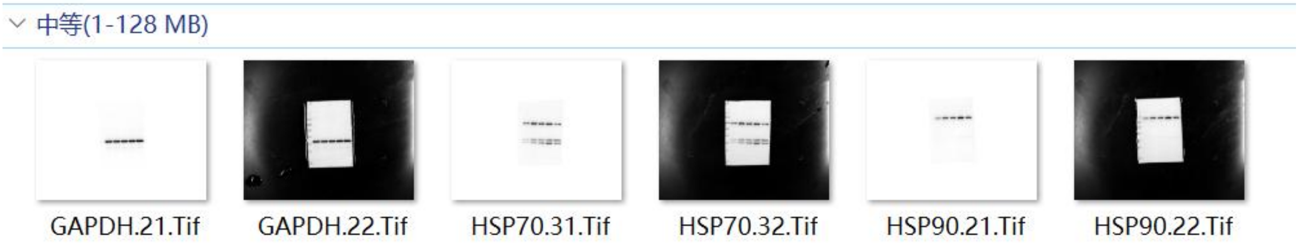
中等(1-128 MB)
GAPDH.21.Tif
GAPDH.22.Tif
HSP70.31.Tif
HSP70.32.Tif
HSP90.21.Tif
HSP90.22.Tif

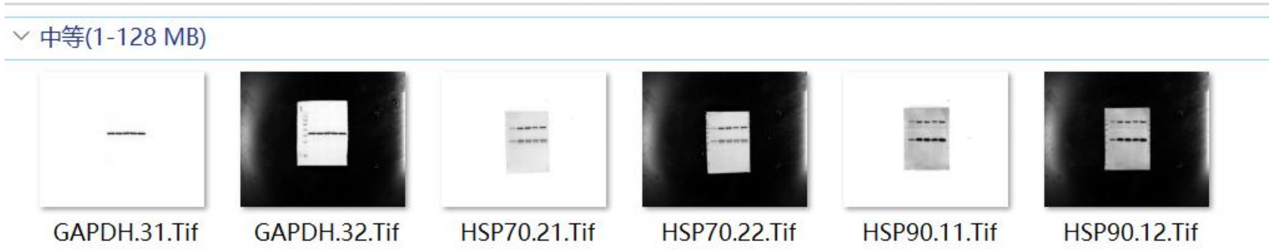
中等(1-128 MB)
GAPDH.31.Tif
GAPDH.32.Tif
HSP70.21.Tif
HSP70.22.Tif
HSP90.11.Tif
HSP90.12.Tif
